# The Inner Nuclear Membrane Protein Nemp1 Is a New Type of RanGTP-Binding Protein in Eukaryotes

**DOI:** 10.1371/journal.pone.0127271

**Published:** 2015-05-06

**Authors:** Takashi Shibano, Hiroshi Mamada, Fumihiko Hakuno, Shin-Ichiro Takahashi, Masanori Taira

**Affiliations:** 1 Department of Biological Sciences, Graduate School of Science, The University of Tokyo, 7-3-1 Hongo, Bunkyo-ku, Tokyo, 113–0033, Japan; 2 Departments of Animal Sciences and Agricultural Biological Chemistry, Graduate School of Agriculture and Life Sciences, The University of Tokyo, 1-1-1 Yayoi, Bunkyo-ku, Tokyo, 113–8657, Japan; University of Colorado, Boulder, UNITED STATES

## Abstract

The inner nuclear membrane (INM) protein Nemp1/TMEM194A has previously been suggested to be involved in eye development in *Xenopus*, and contains two evolutionarily conserved sequences in the transmembrane domains (TMs) and the C-terminal region, named region A and region B, respectively. To elucidate the molecular nature of Nemp1, we analyzed its interacting proteins through those conserved regions. First, we found that Nemp1 interacts with itself and lamin through the TMs and region A, respectively. Colocalization of Nemp1 and lamin at the INM suggests that the interaction with lamin participates in the INM localization of Nemp1. Secondly, through yeast two-hybrid screening using region B as bait, we identified the small GTPase Ran as a probable Nemp1-binding partner. GST pulldown and co-immunoprecipitation assays using region B and Ran mutants revealed that region B binds directly to the GTP-bound Ran through its effector domain. Immunostaining experiments using transfected COS-7 cells revealed that full-length Nemp1 recruits Ran near the nuclear envelope, suggesting a role for Nemp1 in the accumulation of RanGTP at the nuclear periphery. At the neurula-to-tailbud stages of *Xenopus *embryos, *nemp1 *expression overlapped with *ran *in several regions including the eye vesicles. Co-knockdown using antisense morpholino oligos for *nemp1* and *ran* caused reduction of cell densities and severe eye defects more strongly than either single knockdown alone, suggesting their functional interaction. Finally we show that *Arabidopsis thaliana *Nemp1-orthologous proteins interact with *A*. *thaliana *Ran, suggesting their evolutionally conserved physical and functional interactions possibly in basic cellular functions including nuclear transportation. Taken together, we conclude that Nemp1 represents a new type of RanGTP-binding protein.

## Introduction

The nuclear envelope (NE) is not only the boundary that separates the nuclear and cytoplasmic compartments of eukaryotic cells but it also plays regulatory roles in chromatin organization and gene expression through its nucleoplasmic surface [1]. The NE is composed of double nuclear membranes, nuclear pore complexes (NPCs), and a fibrous protein meshwork called the nuclear lamina. The regulatory role of the NE is mainly attributed to NPC proteins and the inner nuclear membrane (INM) proteins [2]. To further elucidate the role of the NE, it is important to identify and characterize INM proteins, because a large number of putative integral NE proteins remain to be analyzed [3].

INM proteins such as Emerin and MAN1 have been shown to bind to lamins and hence reside on the INM [2]. In terms of the function of these INM proteins, Emerin and MAN1 contain LEM domains that interact with the BAF (barrier to autointegration factor) to anchor chromatin to the NE. They also bind to signal transducers at the INM to modulate BMP/TGF-β and Wnt signaling. For example, MAN1 binds to R-Smad and attenuates BMP/TGF-β signaling [4], and Emerin binds to β-catenin and down-regulates Wnt signaling [5]. Thus, INM proteins play regulatory roles in signal transduction in addition to gene regulation, chromatin organization, and NE formation [6].

NPCs mediate the bidirectional transport of proteins and RNAs across the NE. Nuclear transport proteins, such as importin β/karyopherin β, exportin 1/Crm1, and the small GTPase Ran facilitate the transport of proteins through NPCs [7]. Ran exists in a GTP-bound (RanGTP) and a GDP-bound (RanGDP) state, which are enriched in the nucleus and the cytoplasm, respectively. Their differential localizations are maintained by RCC1 (the nucleotide exchange factor for Ran), which binds to the chromatin in the nucleus, and by RanGAP1 (the Ran GTPase activating protein) in the cytoplasm. Importins and exportins function as transporters for various cargos. For example, transcription factors that can interact with importin β are transported as cargos into the nucleus. In the nucleus, they are dissociated from importin β upon the binding of importin β to RanGTP through the effector domain of Ran. Such nuclear transport mechanisms are conserved between animals and plants [8]. Other than nuclear transportation, Ran is also involved in controlling mitotic checkpoints, spindle assembly, and NE re-assembly through its interactions with importins [9], suggesting that Ran is a crucial factor throughout every stage of the cell cycle.

Recently, we identified a new INM protein, Nemp1 (also known as TMEM194A), which is expressed in the anterior neural plate in *Xenopus* [10]. Nemp1 contains an evolutionarily conserved region A within its transmembrane domains (TMs) and region B within its C-terminal region, but does not contain any known domains. We have shown that (i) Nemp1 is localized to the INM; (ii) region B faces the nucleoplasm and binds to BAF through a BAF binding site (BBS); (iii) both overexpression and knockdown of Nemp1 in *Xenopus* embryos reduce the expression of early eye-specific genes, resulting in severe eye defects; and (iv) Nemp1 activity requires regions A, a Lys-Arg-rich (KR) sequence, and region B [10]. Thus, our data suggest that a proper level of Nemp1 at the INM is required for eye development. However, the molecular function of Nemp1 remains to be clarified.

In this study, we analyzed the molecular nature of Nemp1 in terms of the functional roles of region A, the KR sequence, and region B using *Xenopus laevis* and mouse (*Mus musculus*) Nemp1, designated as Xl_Nemp1 and Mm_Nemp1, respectively. To the best of our knowledge, our study is the first report Nemp1 as a new type of Ran-binding protein that exhibits a fundamental cellular function.

## Materials and Methods

### Ethics statement

The work in this paper was conducted using protocols approved by the Animal Care and Use Committee of the University of Tokyo (permit number: 01–13).

### cDNA cloning and plasmid constructs

A full-length cDNA clone (attBpBC-mKIAA0286) of *Mus musculus* (*Mm*) Nemp1 (*Mm_Nemp1*) (accession no. NM_001113211) was obtained from the Kazusa DNA Research Institute. *Mm_ran* (NM_009391) and *Xenopus laevis* (*Xl*) *ran* (NM_001086713) were isolated from the mouse 11-day embryo MatchMaker cDNA library (Clontech) and *Xenopus* total RNA at the neurula stages, respectively. *Arabidopsis thaliana* (*At*) *nemp* genes (NM_102639; NM_001037091; NM_114844) and *At_ran2* (NM_122009) were isolated from *Arabidopsis* total RNA (a gift from Dr. S. Sawa). Plasmid constructs were made with HA, Myc, and FLAG-tagged vectors, which were derived from pCSf107mT [11] and pCS2+. Two-round PCR-based mutagenesis was performed for making point-mutated, deleted, or chimeric constructs. All constructs and vectors used for this study are listed in S1 Table.

### Yeast two-hybrid screening assay

The yeast MatchMaker Two-Hybrid System (Clontech) was used to screen the mouse 11-day embryo MatchMaker cDNA library using Mm_Bt (334 to 437a.a of Mm_Nemp1) as bait. The bait plasmid pGBKT7-Mm_Nemp1_Bt and the cDNA library were sequentially transformed into the yeast strain AH109. Transformants (9 × 10^6^) were plated and screened on 100 mm-diameter plates with medium lacking leucine, tryptophan, and adenine. Colonies were picked and checked for ß-galactosidase production by using a filter assay with 5-bromo-4-chloro-3-indolyl-ß-D-galactopyranoside. Plasmid purification was done from the positive clones, and a second round of interaction screening was performed to confirm the interactions. The inserts from the positive clones were sequenced.

### Microinjection experiments using *Xenopus* embryos

Fertilization and manipulation of *Xenopus laevis* embryos and microinjection of mRNA or morpholino oligo (MO) were carried out as described previously [10]. Embryos were staged according to the criteria of Nieuwkoop and Faber [12]. Antisense MOs for *Xl_nemp1* (*nemp1*MOs) [10] or *Xl_ran* (*ran*MO) were obtained from Gene Tools LLC. *ran*MO is complementary to the sequence encompassing the translation start sites of both homoeologs of *ran* (*Xl_ran-a*: NM_001086713 and *Xl_ran-b*: NM_001135075) (5’-CTTGAGGTTCTCCTTGGGCTGCCAT-3’). Standard control MO (stdMO; Gene Tools LLC) was used as negative control. MOs were dissolved in water and heated at 65 °C for 10 min before use. mRNAs or MOs were injected into a dorsoanimal blastomere at the 4 cell stage, in which the injected area was fated to the anterior neural plate. FITC-dextran (50 ng/embryo) was used as a tracer.

### Whole-mount *in situ* hybridization

Whole-mount in situ hybridization (WISH) was performed according to Harland [13]. Antisense *Xl_ran* RNA probes were transcribed with T7 RNA polymerase from SalI-linearized pGEM-T-Xl_ran.

### Purification of recombinant proteins and GST pulldown assays

GST fusion constructs for Mm_Bt (GST-Mm_Bt) and Myc-tagged Mm_RanQ69L (Myc-RanQ69L) were made using pGEX6Pmcs. Purification of GST-fusion proteins and cleavage of a GST portion from GST-Ran were carried out as described previously [14]. Loading of GTP to recombinant RanQ69L was carried out in binding buffer (20 mm Tris-HCl, pH 8.0, 50 mm NaCl, 2.5 mm MgCl_2_, 0.5% NP-40, 1% BSA and 10% (v/v) glycerol) containing 2 mM GTP by incubating at room temperature for 30 min in a final volume of 50 μl, then diluting to 250 μl in binding buffer. For GST pulldown assays, GST-Mm_Bt attached to glutathione-Sepharose beads was incubated at 4 °C for 2 h with cell lysate (see below) or with GTP-loaded RanQ69L in 300 μl of binding buffer. The beads were washed 4 times with binding buffer. Pulled down proteins were analyzed by western blotting with FluoroTrans membranes (Pall corporation) and the appropriate antibodies as described [10].

### Co-immunoprecipitation

Co-immunoprecipitation (co-IP) assays were performed essentially as described previously [15], with slight modifications. Injected embryos were collected at the mid blastula stage (stages 8–8.5) or the late blastula stage (stage 9), and homogenized in lysis buffer A (20 mM Tris-HCl, pH 8.0, 5 mM EDTA, 10% glycerol, 0.1% NP-40, 8 mM DTT, 40 μg/ml leupeptin, 20 μg/ml aprotinin, 1 mM PMSF) for a region B containing region or lysis buffer B (50 mM Tris-HCl, pH 7.5, 5 mM EDTA, 100 mM NaCl, 0.5% NP-40, 40 μg/ml leupeptin, 20 μg/ml aprotinin, 1 mM PMSF) for full-length Nemp1. Equivalent amounts of lysates were incubated with the appropriate antibody for 1 h at 4 °C, then added with 40 μl of protein G-agarose beads (Roche), and incubated for another 1.5 h at 4 °C. The beads were washed 4 times with the same lysis buffer, added with SDS sample buffer, and boiled to elute bound proteins. Eluates were analyzed by western blotting.

### Immunofluorescence microscopy

Preparation of COS-7 cells transfected with plasmid DNA, and confocal microscopic analysis with LSM Pascal (Zeiss) were performed as described [10]. Immunostaining was performed using mouse anti-Myc 9E10, mouse anti-HA 12CA5, rabbit anti-HA Y-11 (Santa Cruz), mouse anti-pan lamin (X67, X167, X233) (Abcam) and anti-Nup153 QE5 (Abcam) antibodies as primary antibody and Alexa Fluor 488-, Alexa Fluor 555-, and Alexa 546-conjugated antibodies (Molecular Probes) as secondary antibody. Nuclei were stained with SytoxGreen (Molecular Probes). For co-immunostaining with lamin, transfected cells are fixed in methanol at -20 °C.

### In vitro alkaline phosphatase assays

When calf intestine phosphatase (CIAP) (New England Biolabs: NEB) was used, *Xenopus* embryos overexpressing HA-tagged Xl_Nemp1 (Xl_Nemp1-HA) were lysed in lysis buffer A. Lysates were incubated with anti-HA antibody at 4 °C for 1 h, then added with protein G-agarose beads, and incubated for another 1.5 h. The beads were washed 3 times with lysis buffer A, once with NEBuffer 3 (NEB), and incubated in NEBuffer 3 containing 0.5 u/ml of CIAP for 3 h at room temperature. When λ protein phosphatase (NEB) was used, *Xenopus* embryos overexpressing mouse Nemp1-HA (Mm_Nemp1-HA) were lysed in lysis buffer A without EDTA. Lysates were incubated with λ protein phosphatase in NEBuffer for Protein MetalloPhosphatases (NEB) for 45 min at 30 °C. Treated samples were analyzed by western blotting with anti-HA antibody.

### Measuring cell densities and ratios of mitotic cells in *Xenopus* embryos

MOs and FITC-dextran (50 ng/embryo) as a tracer were injected into the dorsoanimal region of four-cell-stage embryos. Injected embryos were fixed at the late gastrula to early neurula stages (stages12.5–13) and immunostained using rabbit anti-phospho-Histone H3 (Ser10) antibody (Millipore) as primary antibody and Alexa Fluor 555-conjugated antibody (Molecular Probes) as secondary antibody, as described previously [11]. Nuclei were stained with DAPI. Confocal microscopic analyses were performed with LSM 710 (Zeiss). Five embryos from each experimental group were used for counting the number of nuclei and pH3-positive nuclei in more than two separate regions (a total area was more than 0.1 mm^2^) of MO-injected and FITC-positive regions of each embryo. For rescue experiments, embryos were co-injected with MOs and *nemp1*, *ran*, or *globin* (negative control) mRNA together with EGFP-HA mRNA (200 pg/embryo) as a tracer, fixed at stages12.5–13, stained with DAPI and anti-HA antibody. Nuclei were counted in EGFP-HA positive areas. The statistical significance (*P*-value) was calculated using Student's or Welch's *t*-test after comparison of the variances of a set of data by *F*-test.

### RT-quantitative PCR (RT-qPCR)

Total RNA was isolated from mock or Mm_Nemp1-HA-transfected COS-7 cells (derived from African green monkey) using Trizol (Life Technologies) and digested with RQ1 DNase (Promega). Quantitative PCR analysis was performed using a StepOnePlus real-time PCR system (Applied Biosystems) with PCR primers for *Nemp1* and *GAPDH* (glyceraldehyde 3-phosphate dehydrogenase). Nemp1-RT primers were designed for conserved sequences in *nemp1* CDSs of mouse, green monkey (*Chlorocebus sabaeus*) (XM_008003729), and human (NM_001130963). Green monkey *GAPDH* (*Cs_GAPDH*) primers were designed for conserved sequences in *gapdh* CDSs of green monkey (XM_007967342) and human (NM_001289745), and used as an internal control. Real-time PCR assays were performed in triplicate using the following primers:

Nemp1-RT-F: 5′-CTCCGAGAATTTTGTAACAGTCC-3′,

Nemp1-RT-R: 5′-ATGCTCCCTAATCCATACTCCTG-3′;

Cs_GAPDH-RT-F: 5′- GAAGGTGAAGGTCGGAGTCAA-3′,

Cs_GAPDH-RT-R: 5′- CATGTAAACCATGTAGTTGAGGTC-3′.

## Results

### Region A of Nemp1 plays a role in its colocalization with lamins

**Fig 1:**
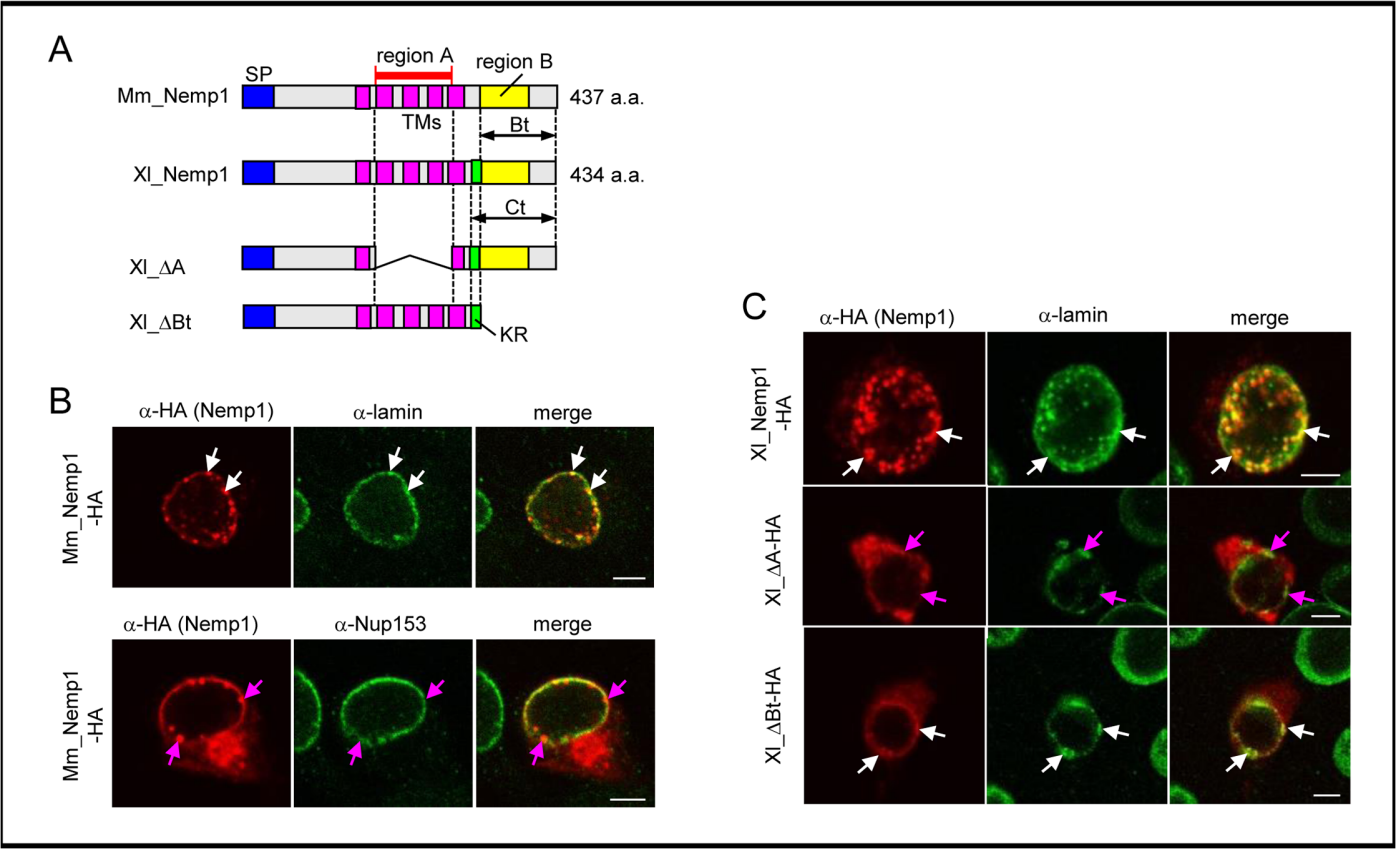
Colocalization of Nemp1 and lamins through region A. (A) The diagram of Xl_Nemp1 and Mm_Nemp1. Xl_Nemp1 but not Mm_Nemp1 contains the KR sequence. Blue, magenta, green, and yellow boxes represent signal peptides (SP), transmembrane domains (TMs), KR sequence, and region B, respectively. (B,C) Confocal analysis was performed using transfected COS-7 cells. (B) Mm_Nemp1-HA with lamin or Nup153. Transfected cells were stained with anti-HA (red) and anti-lamin or anti-Nup153 (green) antibody. Scale bars, 5 μm. (C) Xl_Nemp1-HA or its deletion mutants with lamin. Transfected cells were stained with anti-HA (red) and anti-lamin (green) antibodies. Scale bars, 5 μm.

In this study, we used both Xl_Nemp1 and Mm_Nemp1, in which two conserved regions, region A and region B show 67% and 80% identities, but the KR sequence is unique to Xl_Nemp1 [10] (Fig 1A).

Because region A of Xl_Nemp1 is sufficient for its nuclear envelope (NE) localization [10], we examined the interaction of Nemp1 with lamins and the NPC component Nup153 using specific mouse monoclonal antibodies. Nemp1 was transfected to COS-7 cells, and colocalization was analyzed by confocal analysis. Expression levels of exogenous *Mm_Nemp1-HA* were much higher than those of endogenous *nemp1* in COS-7 cells as assayed by RT-qPCR (S2 Table), implying that the behavior of tagged proteins was not affected by the endogenous protein. The data showed that the punctated staining of Mm_Nemp1-HA significantly colocalized with lamins at the NE (Fig 1B; upper panels, indicated by white arrows) but not as significantly with Nup153 at the NPC (Fig 1B; lower panels, indicated by magenta arrows). Using deletion constructs of Xl_Nemp1, we tested which region of Nemp1 is required for its colocalization with lamins and found that Xl_ΔBt-HA, but not Xl_ΔA-HA, colocalized with lamins (Fig 1C). These data suggest that Nemp1 colocalizes with lamins through region A.

We have previously suggested that the KR sequence is a nuclear localization signal (NLS), which is required for nuclear localization of the C terminal region (Ct; see Fig 1A) [10]. To examine NLS function, the KR sequence was fused to GST-mRFP-HA, which alone cannot be transported into the nucleus. In COS-7 cells, GST-mRFP-HA fused with KR (KRa or KRb from the homoeologs) or SV40NLS as a positive control exclusively localized to nuclei, but KRa(ΔR) or KRm (from Mm_Nemp1) did not. These data clearly indicate that the KR sequence functions as a NLS (S1 Fig A). On the other hand, although the KR sequence is not present in Mm_Nemp1, Mm_Bt (the region B plus its downstream region; see Fig 1A) was localized to the nucleus (S1 Fig B), which is different from Xl_Bt [10], suggesting the possibility that Mm_Bt itself has a cryptic NLS. Therefore, to test this possibility, Mm_Bt was fused to GST-mRFP-HA. This GST-mRFP-Mm_Bt-HA protein exhibited, though in a part of cells, nuclear localization (S1 Fig B), implying that Mm_Bt can exhibit NLS function under some conditions. These data suggest the possibility that the KR sequence in Xl_Nemp1 and the C-terminal region in Mm_Nemp1 as well as region A for association with lamins participate in the INM localization of Nemp1.

### Nemp1 oligomerizes with itself and INM proteins thorough TMs

**Fig 2:**
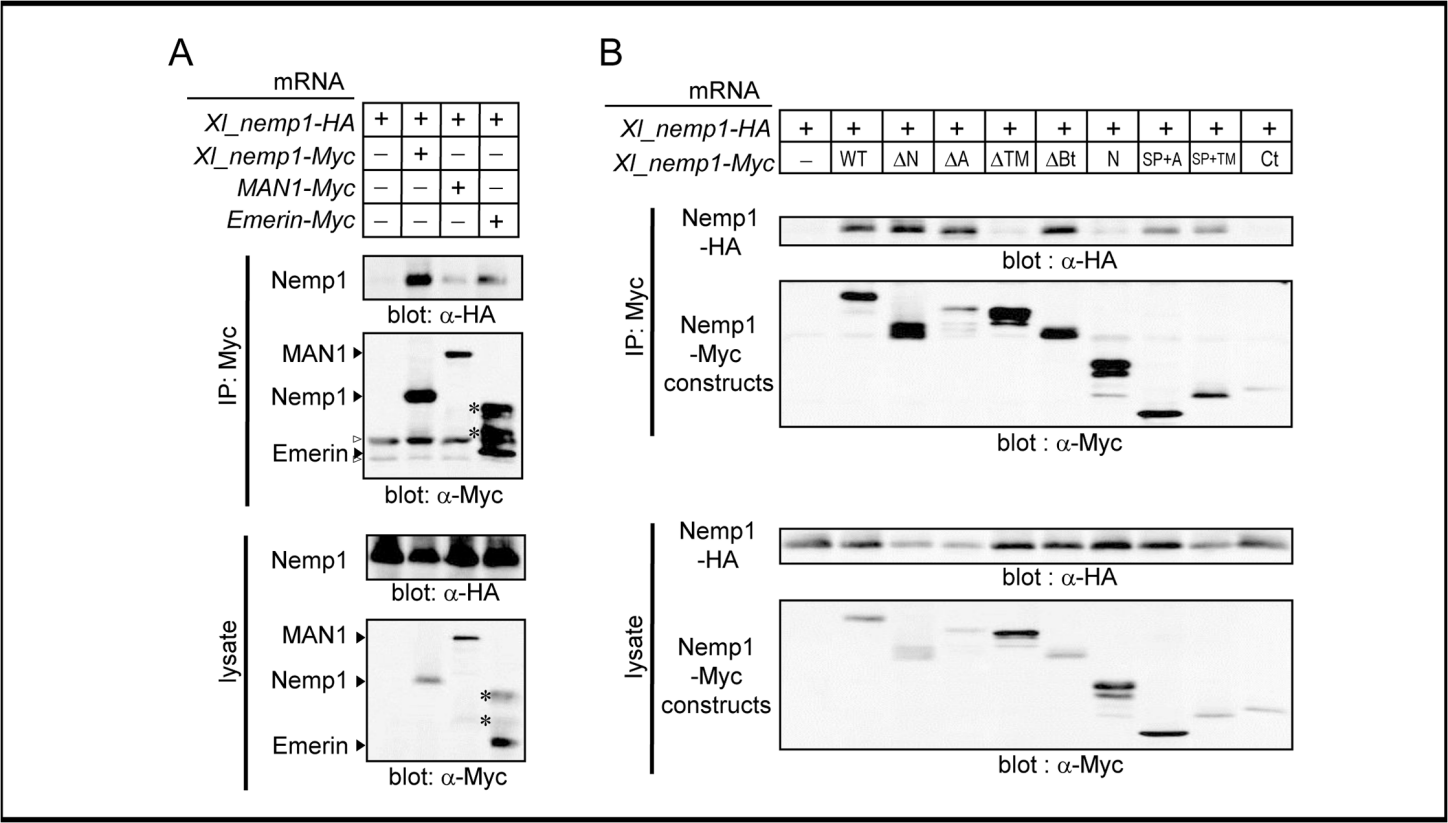
Oligomerization of Nemp1 through the TMs. A. Co-IP of Xl_Nemp1 with Xl_Nemp1 itself, MAN1, or Emerin. Xl_Nemp1-HA mRNA was coinjected into the animal pole region of two cell stage *Xenopus* embryos with mRNA for Xl_Nemp1-Myc, XMAN1-Myc, or Hs_emerin-Myc. Injected embryos were collected at the late blastula stage (stage 9) and lysed with lysis buffer A. Black arrowheads, expected product bands; white arrowheads, immunoglobulin bands; asterisks, shifted bands of Emerin due to phosphorylation [40]. B. Co-IP of Nemp1 with its deletion constructs. mRNA for Xl_Nemp1-HA was injected into *Xenopus* embryos with mRNA for deletion constructs of Xl_Nemp1-Myc. Deletion constructs of Nemp1, ΔN, ΔA, ΔTM, ΔB, N, SP+A, SP+B, and Ct (Δ, deleted; N, the N-terminal region; A, region A; TM, transmembrane domains; B, region B; SP, signal peptide; Ct, the C-terminal region; S2 Fig for diagrams) were used (see [10] for more detail). After immunoprecipitation against Myc, western blotting was performed with anti-Myc or-HA antibody as indicated below each panel.

The INM proteins MAN1 and Emerin have been shown to be associated with each other in vitro [16]. Therefore, we examined the interaction of Nemp1 with MAN1 and Emerin, and with itself by co-IP assays using embryos overexpressing HA-tagged Xl_Nemp1 with either Myc-tagged Xl_Nemp1, MAN1, or Emerin (Fig 2A). We found that Xl_Nemp1 forms a complex with itself and to a lesser extent with MAN1 or Emerin (Fig 2A). Deletion analysis revealed that HA-tagged WT, ΔN, ΔA, ΔB, SP+A, and SP+TM but not ΔTM, N, or Ct (see S2 Fig for structures of constructs) were coimmunoprecipitated with Myc-tagged WT, indicating that the TMs are both required and sufficient for the oligomerization of Nemp1 (Fig 2B). The punctated staining of tagged Nemp1 at the nuclear membrane might reflect the oligomerization ability of Nemp1 (see Fig 1B). These data suggest that Nemp1 complexes could be formed through the TMs in the NE and perhaps the ER, and might directly associate with MAN1 and Emerin or indirectly through the nuclear lamina because all three can associate with lamin.

### The region B of Nemp1 directly binds to Ran

**Fig 3:**
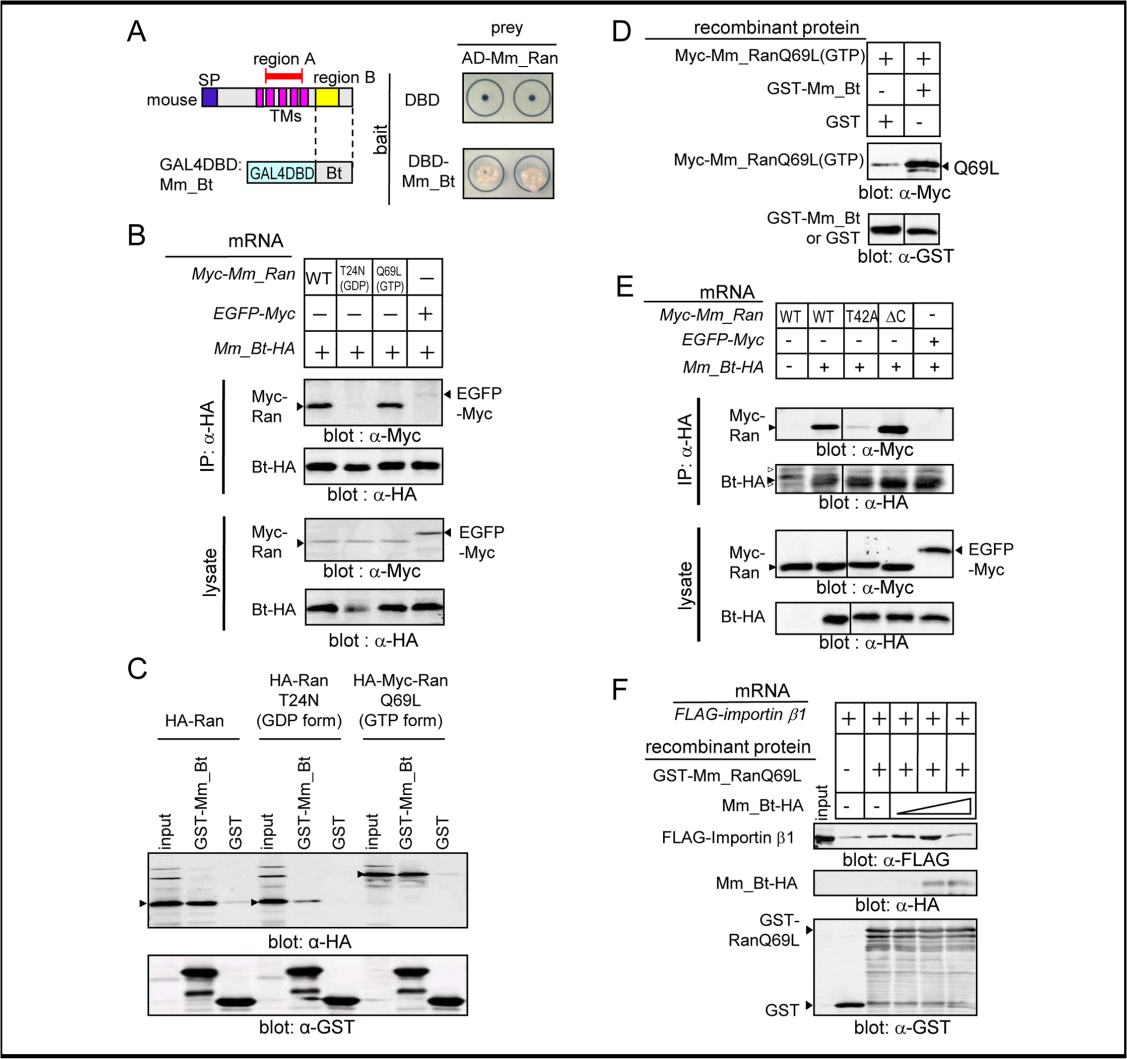
Interaction of region B with RanGTP. A. Yeast two hybrid screening. Left panel, schematic representation of the bait, the Bt region of Mm_Nemp1 (DBD-Mm_Bt). Right panels, colony formation (in duplicate) of yeast AH109 cells transformed with DBD (upper) or DBD-Mm_Bt (lower) with AD-Mm_Ran on plates lacking tryptophan, leucine, and adenine. DBD, the DNA binding domain of Gal4; AD, the activation domain of Gal4. B. Co-IP of region B with Ran or its mutants using *Xenopus* embryos. mRNA for HA-tagged Mm_Bt was coinjected into *Xenopus* embryos with mRNA for a Myc-tagged construct of Mm_Ran, the RanGDP form mutant T24N, the RanGTP form mutant Q69L, or EGFP. Experimental conditions were the same as in Fig 2. C. GST pulldown assays using *Xenopus* embryo lysates. Purified GST or GST-Mm_Bt protein absorbed onto glutathione-Sepharose beads were incubated with lysates of *Xenopus* embryos, which had been injected with mRNA for HA-Mm_Ran, HA-T24N, or HA-Myc-Q69L (500 pg/embryo). Proteins bound to the beads were analyzed by western blotting. D. In vitro binding assays with recombinant proteins, Myc-Mm_RanQ69L(GTP) and GST-Mm_Bt. Purified GST-Mm_Bt or GST (2.8 μg) was incubated with purified Myc-RanQ69L (5 μg), which had been loaded with 2 mM GTP in the binding buffer. E. Co-IP of Mm_Ran mutants T42A and ΔC with Mm_Bt-HA using *Xenopus* embryos. mRNA for HA-tagged Mm_Bt was coinjected into *Xenopus* embryos with mRNA for Myc-tagged Mm_Ran, or its mutants (T42A or ΔC). Unnecessary lanes were removed from a single blot. F. GST pulldown assays of GST-Mm_RanQ69L, importin β and Mm_Bt. *Xenopus* embryos were injected with mRNA for FLAG-tagged importin β. Lysates were added with 0.1, 1, 10 μg of recombinant Mm_Bt and glutathione beads absorbed 10 μg of GST-RanQ69L. Western blotting was performed with antibodies as indicated below each panel. Arrowheads, expected product bands.

Because the previous study has shown that region B faces the nucleoplasm and is required for the eye-reducing activity of Nemp1 in *Xenopus* embryos [10], we searched for region B-interacting proteins using the yeast two-hybrid system. We subcloned Mm_Bt as bait (Fig 3A) for screening a mouse embryonic cDNA library. As a result, we identified several candidate proteins, such as Ran and Ubc9 (not shown), which consistently interacted with Mm_Bt in yeast (Fig 3A). To assess the interaction of Nemp1 with Ran in the *Xenopus* embryo and also in vitro, we performed co-IP and GST pulldown assays using HA- or Myc-tagged proteins. In parallel, we also tested whether Nemp1 binds to either RanGTP or RanGDP using the GTP- and GDP-bound mutant forms RanQ69L and RanT24N, respectively. For co-IP analysis, *Xenopus* embryos were coinjected with mRNAs encoding for Mm_Bt-HA and Myc-Mm_Ran constructs. As shown in Fig 3B, Myc-Ran (WT) and Myc-RanQ69L (GTP-bound form mutant) but not Myc-RanT24N (GDP-bound form mutant) coimmunoprecipitated with Mm_Bt-HA, suggesting that Nemp1 specifically forms a complex with RanGTP through region B in the embryo. Similarly in GST pulldown analysis, HA-Ran and HA-Myc-RanQ69L (GTP form) but not HA-RanT24N (GDP form) from embryonic lysates were pulled down by recombinant GST-Mm_Bt that was purified from bacterial lysates (Fig 3C), indicating that Mm_Bt specifically interacts with RanGTP. To analyze direct interactions, we bacterially synthesized and purified recombinant Myc-RanQ69L by cleaving the GST moiety. Fig 3D shows that Myc-RanQ69L was pulled down by GST-Mm_Bt in comparison with GST alone, demonstrating the direct interaction between Mm_Bt and RanQ69L (GTP form).

We then examined the region of RanGTP that binds to region B. RanGTP is known to bind to both importin β and RanBP1. These interactions are disrupted in Mm_RanT42A, which has a point mutation in its effector domain [17]. In addition, the interaction between RanGTP and RanBP1 is abolished in Mm_RanΔC, which lacks the highly conserved acidic C-terminal tail of Ran (the DEDDDL sequence) [18]. Therefore, to examine whether these regions of Ran interact with Nemp1, we performed co-IP assays using these two mutant constructs. Fig 3E shows that the ΔC mutant but not the T42A mutant co-immunoprecipitates with Mm_Bt, suggesting that region B, similarly to importin β interacts with the effector domain of Ran. Therefore, we next tested whether region B competes for the interaction between importin β and RanQ69L. As expected, importin β was pulled down with GST-RanQ69L, and this interaction was reduced by the addition of recombinant Mm_Bt protein at a high concentration (Fig 3F). These data suggest that region B directly interacts with the same surface of RanGTP as importin β.

**Fig 4:**
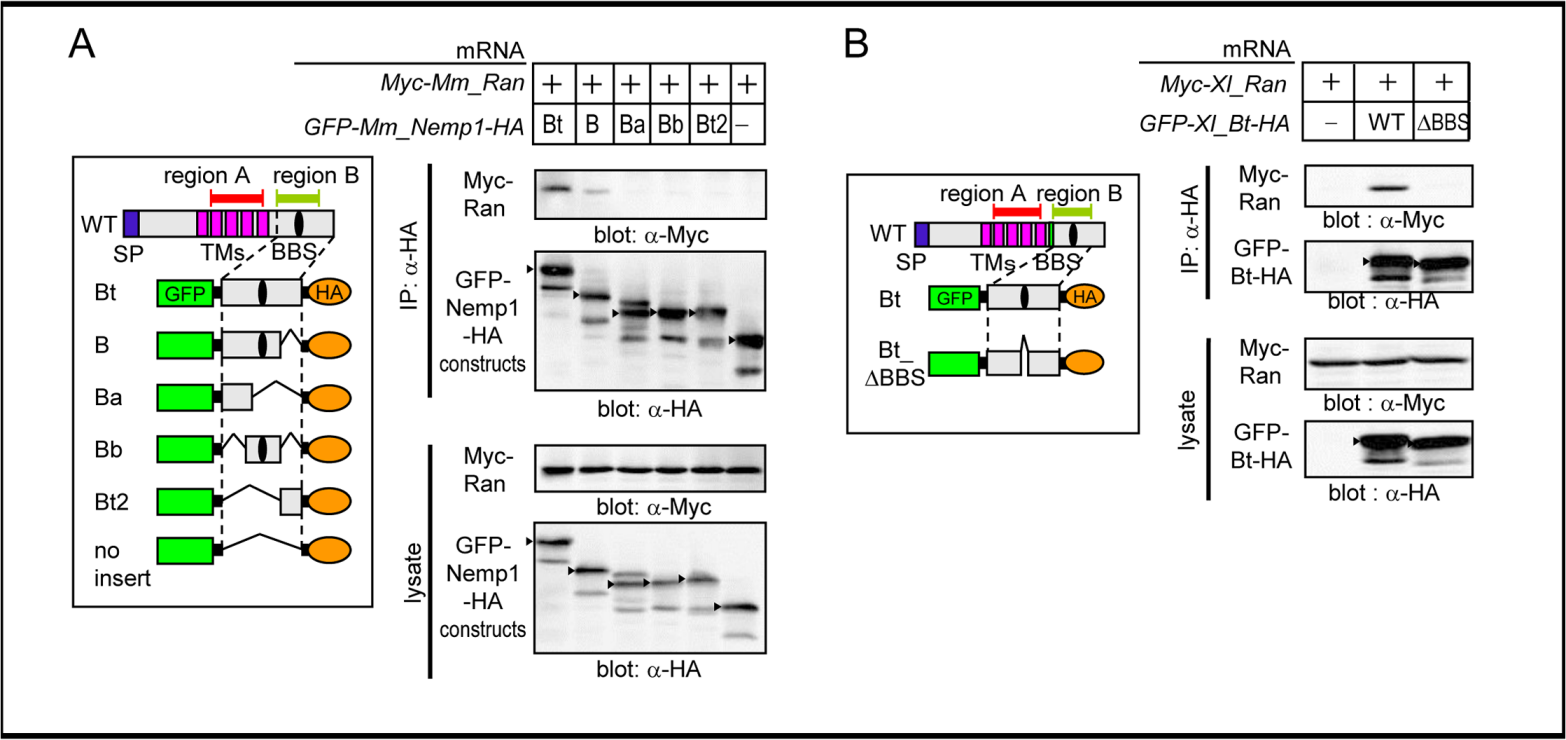
The binding region of region B for Ran. A. Co-IP of Mm_Ran with deletion constructs of Mm_Bt. Left panel, schematic structures of Mm_Bt deletion constructs. Right panels, western blotting of immunoprecipitated proteins or lysates as indicated. mRNA for HA-tagged Mm_Bt constructs was injected with mRNA for Myc-tagged Mm_Ran into *Xenopus* embryos. Arrowheads, expected bands; B. Co-IP of Xl_Ran with Xl_Bt or Xl_Bt_ΔBBS. Left panel, schematic structures of Xl_Bt and Xl_Bt_ΔBBS constructs. Right panels, western blotting of immunoprecipitated proteins or lysates as indicated. Experimental conditions (A, B) were the same as in Fig 3.

To determine a minimal Ran-binding region within the Bt region, we next performed co-IP experiments using HA-tagged deletion constructs of Mm_Bt, which were stabilized by fusing to EGFP. Interactions with Myc-Mm_Ran were detected with Bt and B but not with Ba, Bb, or Bt2 constructs (Fig 4A). Furthermore, the deletion of the BBS (ΔBBS) in Xl_Bt abolished its interaction with Myc-Xl_Ran (Fig 4B). These data suggest that a secondary or ternary structure of region B is required for Ran binding and that the BBS is required for Ran binding as well as BAF binding.

**Fig 5:**
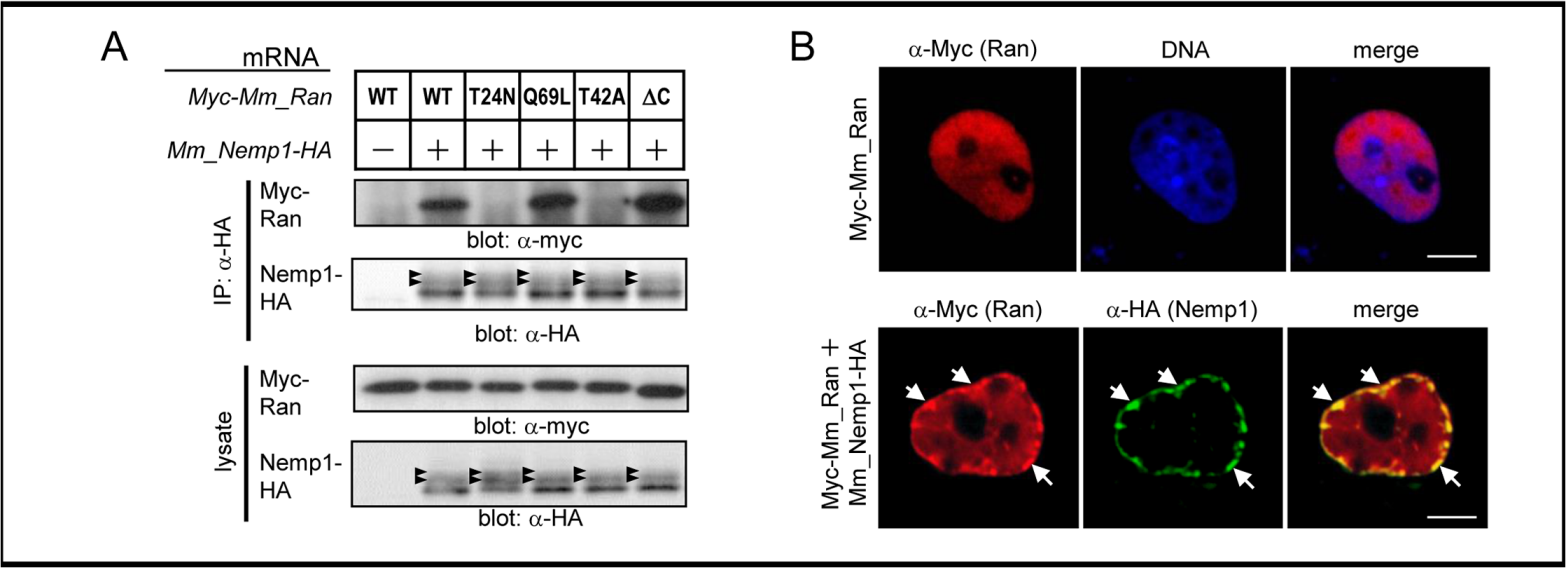
The interaction of Nemp1 with Ran at the NE. (A) Co-IP of Nemp1 with Ran or its mutants using *Xenopus* embryos. mRNA for HA-tagged Mm_Nemp1 was coinjected into *Xenopus* embryos with mRNA for a Myc-tagged construct of Mm_Ran or its mutants (T24N, Q69L, T42A, ΔC). Injected embryos were collected at the mid blastula stage (stage 9) and lysed with lysis buffer B. Black arrowheads, modified forms of Nemp1. This data is the same as lanes 1–6 shown in S3 Fig (B) Colocalization of Ran with Nemp1 at the nuclear periphery. COS-7 cells were transfected with Myc-Mm_Ran (red) with or without Mm_Nemp1-HA (green), and analyzed by confocal analysis. DNA was counterstained with SytoxGreen (blue). Scale bars, 5 μm.

To assess the interaction between Nemp1 and Ran, we used full-length Nemp1 to perform co-IP and confocal microscopic analyses. As shown in Fig 5A, HA-tagged Nemp1 was coimmunoprecipitated with Myc-tagged Ran (WT), RanQ69L, and RanΔC, but not with RanT24N and RanT42A. This data is consistent with that using the Bt region (see Fig 3B and 3E). We next analyzed the localization of Nemp1 and Ran using co-immunostaining of tagged proteins in COS-7 cells by confocal microscopy. Myc-Ran alone was uniformly distributed in the nucleus (Fig 5B; upper panels). By contrast, when coexpressed with Nemp1-HA, Myc-Ran accumulated at the nuclear periphery and colocalized with Nemp1 at the NE (Fig 5B; lower panels). Taken together, our data suggest that Nemp1 at the INM directly interacts with RanGTP in the nucleoplasm.

### Phosphorylation of Nemp1

**Fig 6:**
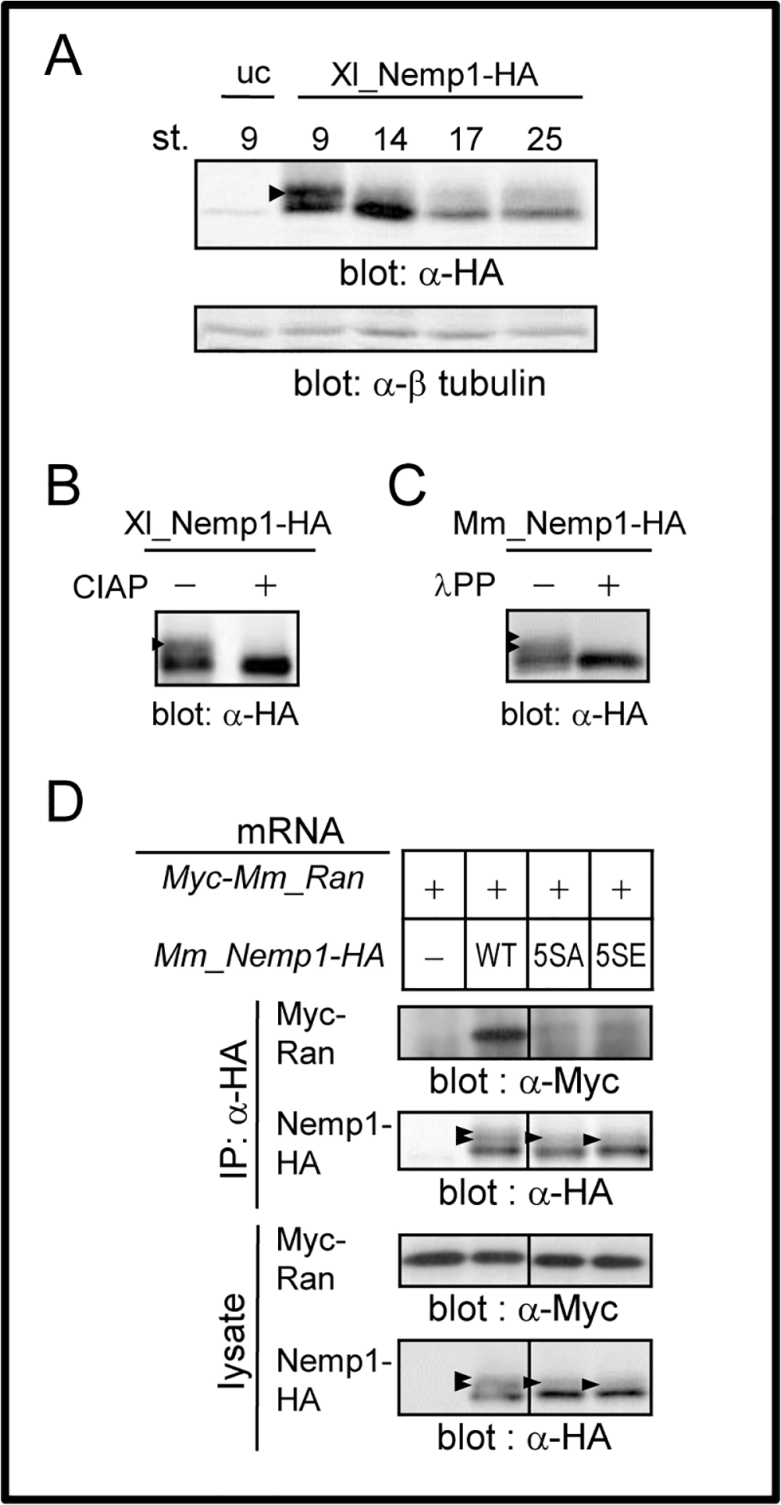
Phosphorylation of Nemp1. A. Developmental analysis for modified Xl_Nemp1. *Xenopus* embryos were injected with mRNA for Xl_Nemp1-HA and collected at the indicated stages (St.). Lysates were subjected to western blotting with anti-HA or β tubulin antibody (loading control). uc, uninjected control. B. In vitro alkaline phosphatase assay of Xl_Nemp1. Lysates were prepared at the late blastula stage (stage 9). Xl_Nemp1-HA was immunoprecipitated by anti-HA antibody and treated with (+) or without (-) calf intestinal alkaline phosphatase (CIAP). C. In vitro alkaline phosphatase assay of Mm_Nemp1. Lysates were prepared at the mid blastula stage (stages 8–8.5), and treated with (+) or without (-) λ protein phosphatase (λPP). D. Co-IP of Mm_Ran with phosphorylation site mutants of Mm_Nemp1. mRNA for Myc-tagged Mm_Ran was injected into *Xenopus* embryos with mRNA for HA-tagged Mm_Nemp1, its alanine mutant (5SA), or its glutamic acid mutant (5SE). Injected embryos were collected at the mid blastula stage and lysed with lysis buffer B. This data is the same as lanes 1, 2, 7, and 8 shown in S3 Fig Black arrowheads, modified forms.

We found that there were shifted bands of Mm_Nemp1 in western blotting of embryo lysates (see Fig 5A and S3 Fig; indicated by asterisks in the panels for Nemp1-HA). We also found in the database from comprehensive analyses of phosphoproteins and phosphorylation sites [19–23], in which human Nemp1 was phosphorylated at multiple sites (Ser368, Ser378, Ser382, Ser424, Ser425). Some of these phosphorylated serines (Ser368, Ser378, Ser382) are located within region B and are evolutionarily conserved among vertebrates, and Ser-378 in the BAF binding sites is also conserved in the vertebrate paralog Nemp2 (see S4 Fig,S7A and S7B Fig). We thus hypothesized that phosphorylation at this site might modulate its interaction with RanGTP. We first examined whether Xl_Nemp1-HA is phosphorylated during early *Xenopus* development when cells are actively divided. Western blotting analysis of embryonic lysates containing the phosphatase inhibitor NaF revealed that shifted bands were strongly detected at the blastula stage (stage 9), and the intensity of these bands was reduced at neurula-to-tailbud stages (stages 14, 17, and 25), suggesting that the modification of Nemp1 occurs in proliferating cells (Fig 6A). Furthermore, phosphatase treatments of immunoprecipitates or lysates abolished shifted bands of both Xl_Nemp1 (Fig 6B) and Mm_Nemp1 (Fig 6C), indicating that modifications of Nemp1 are phosphorylation. To seek phosphorylation sites, we mutated Ser-366, Ser376, Ser380, Ser419, and Ser420 in Mm_Nemp1, which is relevant to the phosphorylated serines in human Nemp1, to Ala or Glu to produce the constructs 5SA or 5SE, respectively. Fig 6D and S3 Fig show that only the upper shifted band was abolished in 5SA and 5SE mutants, suggesting that all or some of these five serine residues function as either phosphorylation sites (probable Ser366-Pro367 and Ser380-Pro381 as Cyclin/Cdk sites) or recognition sites or both, and that other phosphorylation sites exist in Nemp1. Moreover, both the 5SA and 5SE mutations abolished the interaction with Ran, suggesting that all or some of these five serines of Mm_Nemp1 are involved in the interaction with Ran.

### Nemp1 and Ran cooperate to function in early *Xenopus* development

**Fig 7:**
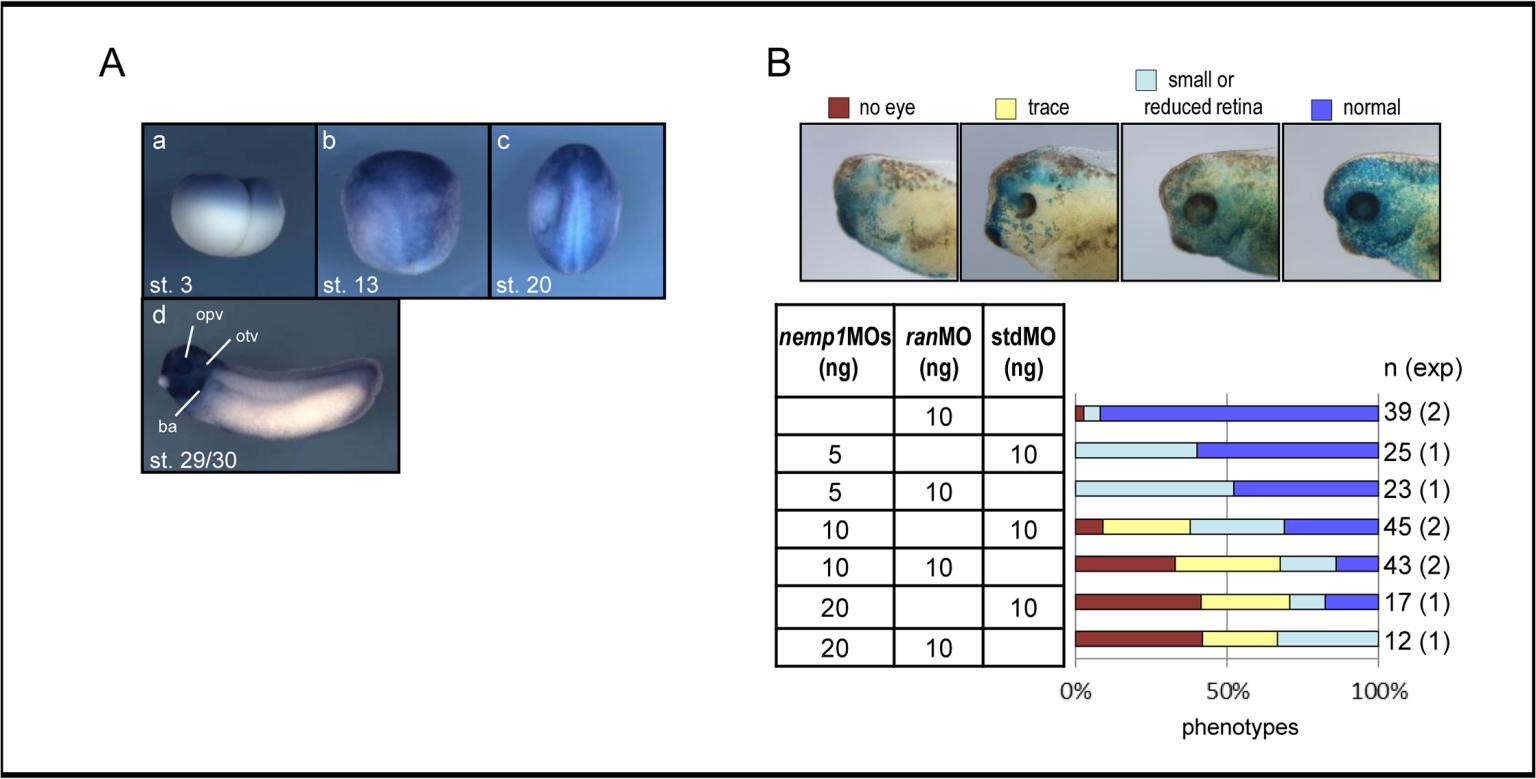
Cooperativity of Nemp1 and Ran in early eye development. A. Spatiotemporal expression of *Xenopus ran* in the early development. Developmental stages are indicated. (a) Lateral view. (b, c) Dorsal view with the anterior side up. (d) Lateral view with the dorsal side up. opv, optic vesicles; otv, otic vesicles; ba, branchial arches. B. Eye defect phenotypes by knockdown of *nemp1* and *ran*. *nemp1*MOs (5–20 ng) and n-βxgal mRNA as a tracer (blue) were injected into the animal pole region of a dorsal blastomere at the four cell stage with *ran*MO or standard control MO (stdMO). Upper panels show eye-defect phenotypes at the tailbud stage (around stage 35) as indicated. The lower bar graph shows percentages of eye defects at tailbud stages. n, the number of embryos examined; exp, the number of independent experiments.

Binding of Nemp1 to Ran prompted us to examine the function of their association in *Xenopus* embryos. Although the expression of *ran* is reported during the development of *Xenopus tropicalis* [24], we re-examined this by WISH with *X*. *laevis* embryos using a short chromogenic reaction to reduce staining intensity. Relatively strong *ran* expression was detected within the animal pole region at the four-cell stage, then in the anterior neural plate at the neurula stage, and within the head region including the otic vesicles, branchial arches, and the tail region at the tailbud stage (Fig 7A). These expression patterns were similar to those of *nemp1* in *Xenopus* embryos [10], consistent with the interaction of Nemp1 with Ran.

To elucidate the cooperative role of Nemp1 and Ran in *Xenopus* eye development, we knocked down both Nemp1 and Ran activities by injecting antisense morpholino oligos (MOs), *nemp1*MOs [10] and *ran*MO. We designed *ran*MO to be complementary to the sequence encompassing the translation start sites of both *X*. *laevis* homoeologs of *ran*. We confirmed that *ran*MO specifically inhibited protein synthesis from Xl_Ran-Myc mRNA containing the MO target sequence but not from Myc-Xl_Ran mRNA without the target (S5 Fig). Injection of *nemp1*MOs or *ran*MO alone exhibited weak activity for inhibiting eye development, whereas the co-knockdown with *nemp1* and *ran* elicited more severe eye defects than either individual knockdown alone (Fig 7B). This data suggests the functional interaction between Nemp1 and Ran.

**Fig 8:**
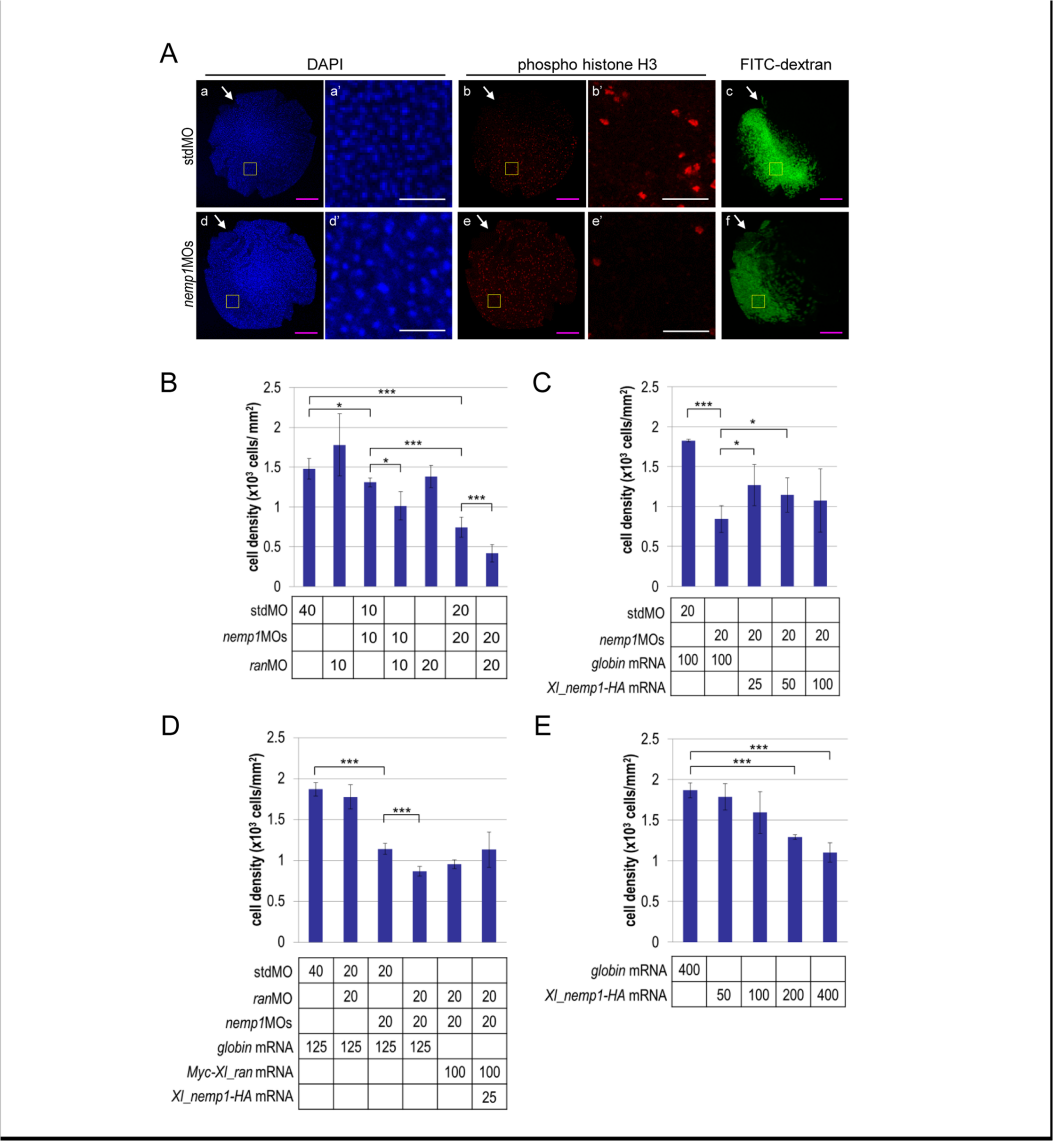
Reduction of cell densities by co-knockdown of *nemp1* and *ran*. (A) A. Effects of *nemp1*MOs on cell densities. Dorsoanterior views (a, b, c, d, e, f) of embryos are shown. Yellow boxes in a, b, d, and e correspond to enlarged areas a’, b’, d’, and e’, respectively. Upper panels, stdMO (40 ng/embryo); lower panels, *nemp1*MOs and stdMO (20 ng each/embryo) (the same experiment as in panel B). Embryos were injected with MOs and FITC-dextran as a tracer, fixed at stages 12.5–13, and immunostained with anti-phospho histone H3 antibody (red). DAPI was used for nuclear staining. White arrowheads, positions of blastopores; magenta scale bars, 500 μm; white scale bars, 100 μm. (B)B. Synergistic effects of *nemp1*MOs and *ran*MOs on cell densities. Combinations of MOs and amounts (ng/embryo) are as indicated. Experiments were repeated three times and similar results were obtained, one of which is presented here. DAPI-stained nuclei were counted in FITC-positive areas. C,D. Rescue of reduced cell density in morphants by mRNA injection. Combinations of MOs and mRNAs as well as amounts of MO (ng/embryo) and mRNA (pg/embryo) are as indicated. Injected embryos were fixed and immunostained using anti–HA antibody. DAPI-stained nuclei were counted in EGFP-HA positive areas. (E) E. Reduction of cell densities by overexpression of Nemp1. Injected mRNA and amount (pg/embryo) are as indicated. DAPI-stained nuclei were counted in EGFP-HA positive areas. *, P<0.05; ***, P<0.005; error bars, standard deviation.

Because *nemp1* and *ran* are expressed in the anterior neural region and eye vesicles, in which cells highly proliferate, we next performed loss-of- and gain-of-function experiments for *nemp1* and *ran* to examine their effects on cell densities and ratios of mitotic cells at the late gastrula to early neurula stages (stages12.5–13) (Fig 8 and S6 Fig). Injection of *nemp1*MOs (10 or 20 ng/embryo) but not stdMO or *ran*MO significantly reduced cell densities (Fig 8A and 8B) and was likely to increase nuclear size (compare panels a’ and d’ in Fig 8A). Co-injection of *ran*MO with *nemp1*MOs further reduced cell densities compared to single knockdowns (Fig 8B). The reduction by *nemp1*MOs was significantly rescued by low doses of *nemp1* mRNA (Fig 8C) and the reduction by both MOs tended to be rescued by *nemp1*and *ran* mRNAs (Fig 8D). These data suggest again the functional synergism between Nemp1 and Ran. Similar to eye phenotypes [10], high doses of *nemp1* mRNA as well as nemp1 MO reduced cell densities (Fig 8E), suggesting that a proper level of Nemp1 is required for normal functions. Supporting the reduction in cell density, single knockdown of *nemp1* or co-knockdown of *nemp1* and *ran* as well as overexpression of *nemp1* by mRNA injection tended to decrease mitotic rates, which were determined using anti-phosphohistone-H3 antibody (S6A and S6B Fig). Thus, it is likely that the reduction in cell density at the neurula stage is caused by the reduction in cell cycle progression by knockdown or overexpression of Nemp1 and Ran. Taken together, the data suggest that *nemp1* and *ran* function cooperatively in proper cell cycle progression and eye development in *Xenopus*.

### Interaction between Nemp and Ran is evolutionally conserved in *Arabidopsis*

**Fig 9:**
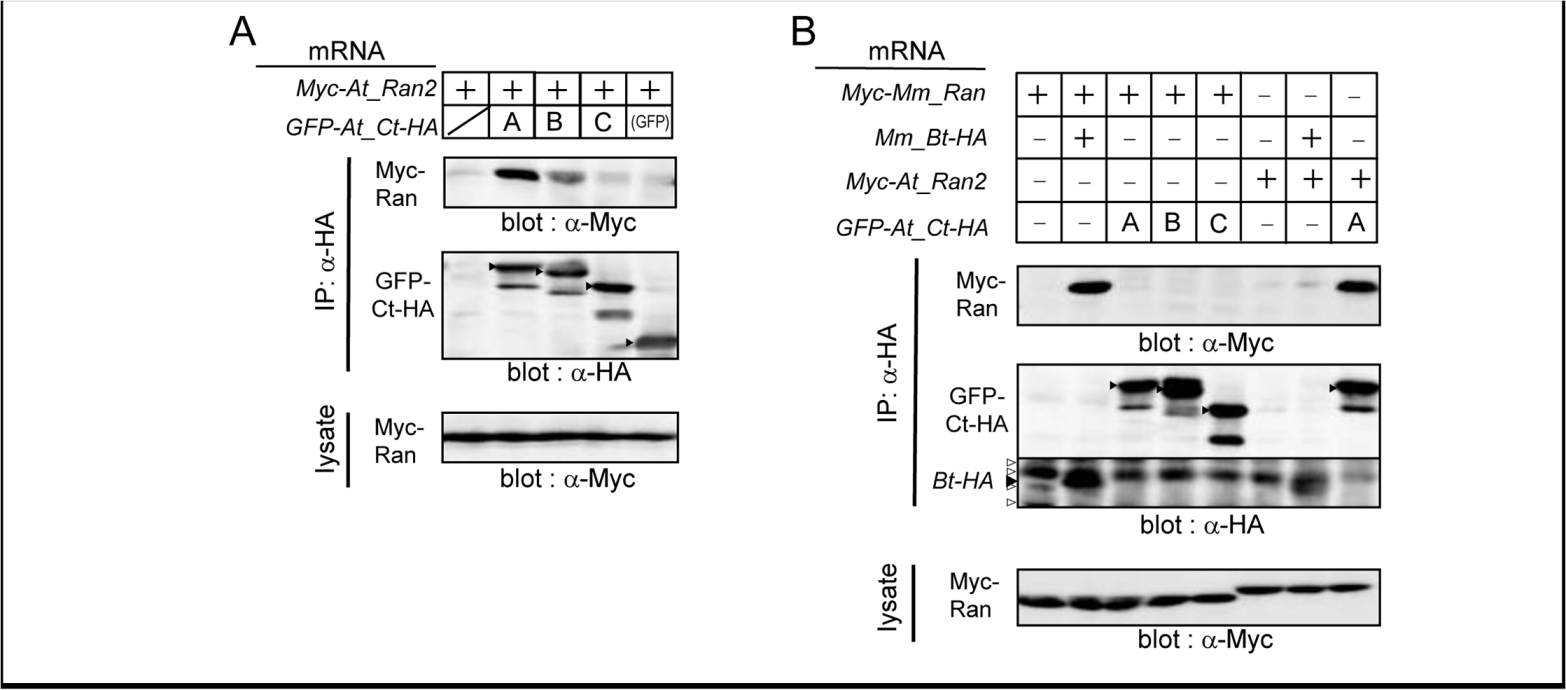
Evolutionary conservation of Ran binding of region B in *Arabidopsis*. A. Co-IP of At_Nemp and At_Ran. Because region B of At_Nemp proteins are not well defined by comparison to vertebrate Nemp1, which is attributed to low amino acid conservation, the entire C-terminal regions downstream of the last TM (named Ct, see S7 Fig C) were used for co-IP experiments. mRNA for GFP-tagged At_Ct constructs were injected with mRNA for Myc-tagged At_Ran2 into *Xenopus* embryos. B. Co-IP of At_Nemp with Mm_Ran or Mm_Nemp1 with At_Ran2. *Xenopus* embryos were coinjected with combinations of mRNAs as indicated. Experimental conditions were the same as in Fig 3. Black arrowhead, expected product bands; white arrowheads, cross-reacted bands. After immunoprecipitation against HA, western blotting was performed with anti-Myc or HA antibody as indicated.

Database search and phylogenetic analysis revealed that Nemp proteins exist not only in eumetazoans including *Nematostella vectensis* (sea anemone) but also in a plant, *Arabidopsis thaliana* (S7A and S7C Fig). To examine whether or not the interaction of Nemp with Ran is conserved in eukaryotes, we performed co-IP assays for the three *Arabidopsis* Nemp proteins (At_Nemp-A, At_Nemp-B, and At_Nemp-C; S7 FigC) and At_Ran2. At_Ran2 is one of the four Ran proteins in *Arabidopsis* and is the most related to vertebrate orthologs (75% amino acid identity). As shown in Fig 9A, At_Ran2 coimmunoprecipitated significantly with the C-terminal region of At_Nemp-A, weakly with those of At_Nemp-B, and barely with At_Nemp-C. Notably, Fig 9B shows that Mm_Bt did not interact with At_Ran2, and conversely, At_Nemps (the Ct regions) did not interact with Mm_Ran, suggesting that Nemp and Ran coevolved to interact with each other. Based on the conserved interaction between Nemp and Ran in eukaryotes, the role of Nemp as a Ran-interacting protein might be related to basic cellular functions, such as the nuclear import system.

## Discussion

A previous study has shown that the signal peptide and TMs are necessary and sufficient for Nemp1 to localize at the NE [10]. The localization of nuclear membrane proteins to the INM is postulated to be facilitated by the following two mechanisms: diffusion-retention and importin α/β-mediated transport. For example, in the former case, MAN1 moves diffusely to the INM from the ER and is retained by the binding of its N-terminal domain to lamins [16,25]. In the latter case, Heh2, a yeast homolog of vertebrate LEM2, contains a canonical NLS-like sequence within its N-terminal nucleoplasmic domain and is transported to the INM by importin α/β complexes [26]. We have shown that Nemp1 colocalizes with lamina through region A (Fig 1), and that the C-terminal region of Nemp1 exhibits nuclear localization activity (S1 Fig). Our data suggest that Nemp1 localizes at the INM via both diffusion-retention and NLS-dependent transport mechanisms.

We have found that Nemp1 specifically interacts with RanGTP via region B (Figs 3 and 4). RanGTP is known to interact with various factors that are associated with nuclear transport and spindle formation, including importins, exportins, RanBP1, and RanBP2. The following two types of RanGTP-binding motifs have been reported: importin β and RanBP1/2 motifs [27,28]. As the conserved motif of region B is different from these two types, Nemp1 might represent a new type of Ran-binding motif. Furthermore, Nemp1 is the first identified nuclear membrane protein reported to bind to RanGTP.

What is the function of Nemp1? We have shown that the coexpression of Mm_Nemp1 promotes the accumulation of Ran at the nuclear envelope (NE) in COS-7 cells (Fig 5B). Based on this observation, it is possible to speculate that the role of Nemp1 is to promote the accumulation of RanGTP at the nuclear periphery. This idea is supported by observations in *C*. *elegans* and *Arabidopsis* that endogenous Ran localizes at the NE during interphase [29,30]. The peripherally biased distribution of RanGTP in the nucleus might be important for efficient dissociation of cargo-importin complexes, which are imported through the NPC and could immediately encounter enriched levels of RanGTP.

Some nuclear lamina proteins (lamins and INM proteins) are reported to be phosphorylated in the prophase of mitosis, resulting in their dysfunction during the NE breakdown. For example, lamin filaments are depolymerized upon phosphorylation of lamin B proteins [31,32], and the LBR (lamin B receptor) is dissociated from chromatin upon phosphorylation of LBR [33]. Similar to these NE proteins, our data suggest the possibility that phosphorylation of Nemp1 occurs in mitosis (Fig 6A,6B and 6C). In addition, serine residues at possible phosphorylation sites are involved in the interaction with Ran (Fig 6D). These data suggest that the interaction of Nemp1 with Ran can be regulated by phosphorylation.

A previous study revealed that Nemp1 interacts with BAF through the BBS and that the BBS is required for the eye-reducing activity of overexpressed Nemp1 [10]. Recently, it was reported that in the absence of DNA, there is no interaction of BAF with BBS-containing proteins, such as CRX and MAN1 [34]. Nemp1 might also indirectly bind to BAF via DNA. In this study, we have shown that the deletion of BBS abolishes the interaction of the Bt region with Ran (Fig 4B). Furthermore, both BAF [10] and Ran accumulate at the nuclear periphery via Nemp1 (Fig 5B). These data suggest that the eye-reducing activity of overexpressed Nemp1 is mediated through its interactions with BAF and Ran. Although Nemp1 is likely to play a role in promoting the accumulation of Ran at the nuclear periphery, the excessive accumulation of Ran as well as of BAF at the nuclear periphery by overexpression of Nemp1 likely perturbs their normal functions. Conversely, the co-knockdown of *nemp1* and *ran* elicits reduction of cell density and eye defects more significantly than the individual knockdown for *nemp1*, supporting their functional interaction.

How is Nemp1 involved in eye development? While there seems to be no direct relationship between the function of Nemp1/Ran and eye development, we propose two possibilities. First, Nemp1 might regulate the nuclear transport of eye-specific transcription factors. In humans, the importin β family consists of 20 members [35], of which importin 13 is known to function in the import of several transcriptional factors, such as Pax6 and Crx, which are important for eye development [36]. Knockdown of *nemp1* reduced the expression of early eye marker genes, *rax* and *pax6* [10]. Therefore, it is conceivable that Nemp1 controls importin13-mediated transport. The second possibility is that Nemp1 is associated with cell proliferation, which is important for eye development. This possibility is based on the fact that knockdown of *nemp1* caused the reduction of cell densities (Fig 8A and 8B), and that expression patterns of *nemp1* and *ran* (Fig 7A) are similar to those of cell cycle regulators, *cyclin D1*, *cyclin E*, and *cdk4* [37]. These data suggest that increased levels of Nemp1 and Ran are necessary for maintaining an actively proliferative state, in which nuclear uptake is more likely to be active than in non-proliferating cells [38].

Our results show that the functional interaction between Nemp1 and Ran is required for proper eye development in *Xenopus* and that their physical interaction is conserved, even in eyeless organisms, such as *C*. *elegans* [39] and plants (Fig 9). Furthermore, *nemp1* and *ran* are co-expressed in tissues other than the eyes in *Xenopus* embryos. Therefore, the interaction between Nemp1 and Ran at the nuclear periphery likely plays important general roles in regulating the nuclear transport of proteins during cellular proliferation and differentiation. We conclude from the results that the inner nuclear membrane protein Nemp1 represents a new type of RanGTP-binding protein and that this interaction might control the nuclear transport of molecules in eukaryotes.

## Supporting Information

S1 Fig NLS function of the *Xenopus* KR sequence and Mm_Bt. (A) Subcellular localization of GST-mRFP fusion constructs for the *Xenopus* KR sequence. Upper panel, schematic representation of GST-mRFP fusion constructs. KRa, KRb, and KRm were derived from the KR of Xl_Nemp1a, Xl_Nemp1b, and the corresponding region of Mm_Nemp1, respectively. KRa(ΔR) is a deletion mutant of KRa. Lower panels, subcellular localization of GST-mRFP fusion constructs. COS-7 cells were transfected with HA-tagged GST-mRFP fusion constructs as indicated, fixed, and stained with anti-HA antibody (red) and SYTOX Green for DNA. Scale bars, 5 μm. (B) Subcellular localization of Mm_Bt and its GST-mRFP- HA construct. COS-7 cells were transfected with the HA-tagged mouse Bt construct (Mm_Bt-HA) or GST-mRFP-Mm_Bt-HA, fixed, and stained with anti-HA antibody (red) and SYTOX Green for DNA. GST-mRFP-Mm_Bt-HA exhibited cytoplasmic localization, but also nuclear localization in some cases. Scale bars, 5 μm. We have previously shown that Myc-tagged Xl_Ct and KR constructs but not Xl_Bt (see Fig 1A) is localized to the nucleus, suggesting NLS function of the KR sequence [10]. Therefore, we systematically examined the nuclear localization activity of KR, using GST-mRFP-HA, which alone cannot be transported into the nucleus because of its large molecular mass (122 kDa as a dimer under the native conditions). GST-mRFP-HA was fused with short peptides related to the KR sequence, and the fusion constructs were analyzed for their ability to localize to the nucleus. As shown in S1 Fig A, GST-mRFP-HA alone was localized in the cytoplasm, whereas the SV40NLS fusion, which served as a positive control, exhibited nuclear localization. Similarly, the KR fusion proteins, KRa and KRb, which were derived from the *Xenopus* homoeologs of Nemp1, Nemp1a and Nemp1b, exhibited nuclear localization, whereas the KRa(ΔR) fusion did not, indicating that both KRa and KRb sequences function as NLSs (S1 Fig B) and that the first Arg residue of the RKIKXKRAK (X is R or L) motif is required for this activity. We also analyzed a short sequence from Mm_Nemp1, whose position corresponds to that of KR in Xl_Nemp1, named KRm, though KRm does not contain a canonical NLS sequence. As expected, KRm did not elicit NLS function (S1 Fig A). However, although Mm_Bt does not have a canonical NLS sequence either, HA-tagged Mm_Bt exhibited nuclear localization (S1 Fig B; upper panels). Therefore, we analyzed NLS function of Mm_Bt suing GST-mRFP-HA, and observed that GST-mRFP-Mm_Bt-HA exhibited weak nuclear localization (middle panels, compare with GST-mRFP-HA negative control), and, in a few cases, it was exclusively localized to the nucleus (lower panels), suggesting that Mm_Bt could have NLS function. Thus, we conclude that the C terminal region of Nemp1 proteins (that is, KR in Xenopus and Bt in mouse) exhibits NLS function.
(TIF)

S2 Fig Diagram of deletion constructs of Xl_Nemp1. These deletion constructs were used in Fig 2B. Blue, signal peptides (SP); magenta, transmembrane domains (TMs); green, KR sequence; yellow, region B.
(TIF)

S3 Fig Co-IP of Nemp1 with Ran using *Xenopus* embryos. This is the original data for Figs 5A and 6D. mRNA for HA-tagged Mm_Nemp1 or its mutants (5SA, 5SE) was coinjected into *Xenopus* embryos with mRNA for a Myc-tagged construct of Mm_Ran (WT) or its mutants (T24N, Q69L, T42A, ΔC). Injected embryos were collected at the mid blastula stage (stages 8–8.5) and lysed with lysis buffer B for Co-IP. Black arrowheads, modified forms of Nemp1-HA. Note that WT Nemp1 has two major modified bands (lane 2) and co-expression with Ran(T24N; a GDP form) enhanced these modifications (lane 3). Also note that the upper modified band disappeared in 5SA and 5SE constructs (lanes 7, 8), suggesting that all or some of these five serine residues are involved in modification (phosphorylation) by functioning as either phosphorylation sites or recognition sites or both, and that there are other phosphorylation sites besides there five serine residues.
(TIF)

S4 Fig Amino acid sequence alignment of Nemp proteins. Only region A (red box) and region B (green box) were aligned for Mm_Nemp1, Mm_Nemp2, Xl_Nemp1b, Dm_Nemp, At_Nemp-A, At_Nemp-B, At_Nemp-C, and Mb_Nemp. Dots, identical amino acid residues; hyphens, gaps; dashed line, DUF2215 domain. The KR sequence and BAF binding sites are colored in yellow as indicated. Blue boxes indicates phosphorylation sites in Mm_Nemp1 and the corresponding serine residues in other species. The serine residues corresponding to Ser-366, Ser-376, and Ser380 (but not Ser419, and Ser420) in Mm_Nemp1 are conserved in Xl_Nemp1. BAF binding sites containing Ser380 are conserved in vertebrate Nemp1 and Nemp2, but not in others. At, *Arabidopsis thaliana*; Dm, *Drosophila melanogaster*; Mb, *Monosiga brevicollis* (choanoflagellate); Mm, *Mus musculus*; Xl, *Xenopus laevis*.
(TIF)

S5 Fig Specificity of ranMO. Nucleotide sequences of Xl_*ran-a*, *b* mRNAs around the initiation codon (underlined), and *ran*MO (upper panel). Western blot analysis of Myc-tagged Xl_Ran fusion protein (lower panel). *ran*MO or stdMO (60 ng) was injected into both blastomeres of two cell stage embryos, and followed by injection with either 200 pg of Xl_Ran-Myc or Myc-Xl_Ran mRNA.-, embryos injected with mRNA alone.
(TIF)

S6 Fig Gain- and loss-of-function experiments for the ratio of mitotic cells. Combinations of injected MOs and mRNAs as well as amounts of MO (ng/embryo) and mRNA (pg/embryo) are as indicated. Experiment conditions are the same as in Fig 8. (A) Reduction of the ratio of mitotic cells by co-knockdown of Nemp1 and Ran. Similar tendencies were obtained from the three experiments and statistically significant differences was observed in one of them. Nuclei stained with DAPI or immunostained for phospho histone H3 were counted in FITC-positive areas. (B) Reduction of the ratio of mitotic cells by overexpression of Nemp1. DAPI-stained nuclei were counted in EGFP-HA positive areas. *, *P*<0.05; ***, *P* <0.005; error bars, standard deviation.
(TIF)

S7 Fig Phylogenetic and syntenic analyses of the Nemp family. A. Phylogenetic analysis. A phylogenetic tree was constructed by the Maximum Likelihood (ML) method using Treefinder with the protein matrix LG after amino acid sequences of the DUF2215 domain in various organisms were aligned using the ClustalW alignment tool with the Gonnet series protein weight matrix (see S4 Fig) and trimmed using trimAl. Values beside nodes show the number of times that a node was supported in 1000 bootstrap pseudoreplication. *Arabidopsis* Nemp homologs (At_Nemp-A, B, and C) serve as outgroups. Note that Nemp is evolutionary conserved from metazoans to choanoflagellates to plants, mainly in the terminal part of region A (see S4 Fig). In vertebrates, a Nemp1 homolog, named TMEM194B or Nemp2, is present in the genome databases of zebrafish, chick, mice, and humans. A de novo phylogenetic tree revealed that Nemp1 and Nemp2 form sister groups in vertebrates (not shown), indicating that Nemp2 is the vertebrate paralog of Nemp1. Abbreviations of species and common names are as follows: plant *Arabidopsis thaliana* (At), Florida lancelet *Branchiostoma floridae* (Bf), nematode *Caenorhabditis elegans* (Ce), ascidian *Ciona intestinalis* (Ci), *Drosophila melanogaster* (Dm), zebrafish *Danio rerio* (Dr), chick *Gallus gallus* (Gg), human *Homo sapiens* (Hs), choanoflagellate *Monosiga brevicollis* (Mb), mouse *Mus musculus* (Mm), sea anemone *Nematostella vectensis* (Nv), African clawed frog *Xenopus laevis* (Xl), and western clawed frog *Xenopus tropicalis* (Xt). Accession numbers of amino acid sequences: Hs_Nemp1, O14524; Mm_Nemp1, Q6ZQE4; Gg_Nemp1, XM_001232566; Xl_Nemp1a, NP_001090391; Xl_Nemp1b, NP_001091224; Xt_Nemp1, NP_001034832; Dr_Nemp1, XP_683418; Hs_Nemp2, A6NFY4; Mm_Nemp2, Q8CB65; Gg_Nemp2, Q5ZJY9; Dr_Nemp2, XP_693037; Bf_Nemp, XP_002585718; Ci_Nemp, AK116477; Sk_Nemp, XP_002741981; Sp_Nemp, XP_001196379; Dm_Nemp, NP_573142; Ce_Nemp, NP_497202; Nv_Nemp, XP_001640959; At_Nemp-A, NM_102639; At_Nemp-B, NM_001037091; At_Nemp-C, NM_114844; Mb_Nemp, XP_001742508. B. Conserved synteny of vertebrate *nemp2* genes. A boat-shape object represents a gene with a direction, in which the tip of boat corresponds to the 3’ end of the gene. Genes indicated with a same color mean orthologous genes, in which white boats indicates unrelated genes. Black boats indicate *nemp2*. Black circles indicate the ends of chromosomes or scaffolds. These maps are drawn based on JGI Metazome data, with some manual editing and corrections. The corresponding synteny maps of *X*. *laevis* (ver. 7.1) and *X*. *tropicalis* (ver. 7.1) suggest that *Xenopus* species do not have *nemp2* orthologs. In addition, EST databases for *X*. *laevis* and *X*. *tropicalis* do not contain *nemp2*-like sequences. C. Diagram of *Arabidopsis* Nemp-A,-B, and-C proteins. According to the *Arabidopsis* genome sequence, typical signal peptide (SP) sequences were not detected in At_Nemp-B and At_Nemp-C. At_Nemp-C is predicted to contain six TMs, but the last two TMs may be a single TM. Colored boxes: blue, signal peptides (SP); magenta, transmembrane domains (TMs); yellow, region B.
(TIF)

S1 Table The list of plasmid constructs used in this paper. (TIF)

S2 Table Comparison of expression levels between endogenous and exogenous Nemp1 mRNAs in DNA-transfected COS-7 cells. (TIF)
