# Supplementary figures and images for: The Inner Nuclear Membrane Protein Nemp1 Is a New Type of RanGTP-Binding Protein in Eukaryotes
Source: PLoS One. 2015 May 6;10(5):e0127271. doi: 10.1371/journal.pone.0127271 (PMC4422613; doi:10.1371/journal.pone.0127271)

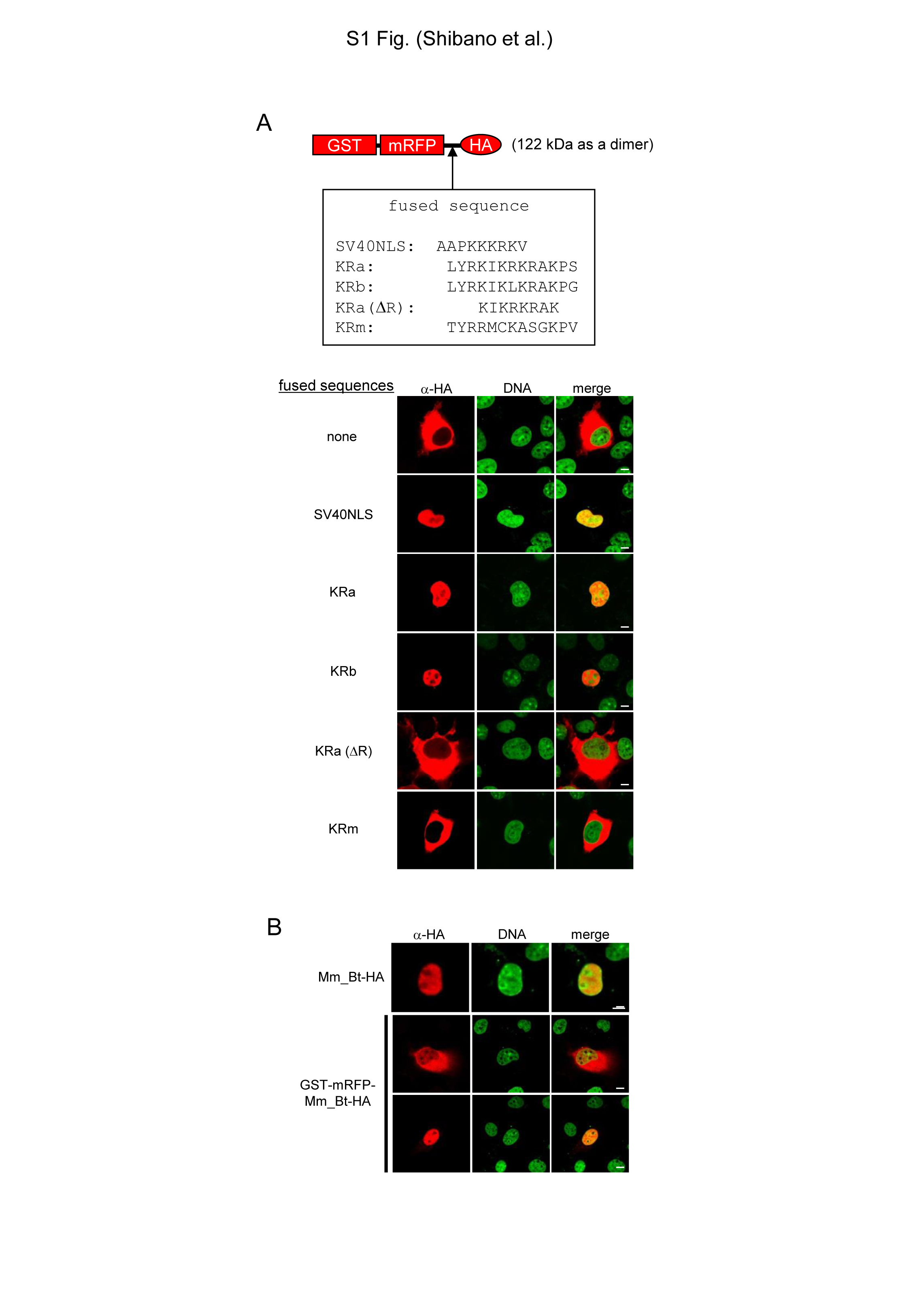

Supplement: S1 Fig — (A) Subcellular localization of GST-mRFP fusion constructs for the Xenopus KR sequence. Upper panel, schematic representation of GST-mRFP fusion constructs. KRa, KRb, and KRm were derived from the KR of Xl_Nemp1a, Xl_Nemp1b, and the corresponding region of Mm_Nemp1, respectively. KRa(ΔR) is a deletion mutant of KRa. Lower panels, subcellular localization of GST-mRFP fusion constructs. COS-7 cells were transfected with HA-tagged GST-mRFP fusion constructs as indicated, fixed, and stained with anti-HA antibody (red) and SYTOX Green for DNA. Scale bars, 5 μm. (B) Subcellular localization of Mm_Bt and its GST-mRFP- HA construct. COS-7 cells were transfected with the HA-tagged mouse Bt construct (Mm_Bt-HA) or GST-mRFP-Mm_Bt-HA, fixed, and stained with anti-HA antibody (red) and SYTOX Green for DNA. GST-mRFP-Mm_Bt-HA exhibited cytoplasmic localization, but also nuclear localization in some cases. Scale bars, 5 μm. We have previously shown that Myc-tagged Xl_Ct and KR constructs but not Xl_Bt (see Fig 1A) is localized to the nucleus, suggesting NLS function of the KR sequence [10]. Therefore, we systematically examined the nuclear localization activity of KR, using GST-mRFP-HA, which alone cannot be transported into the nucleus because of its large molecular mass (122 kDa as a dimer under the native conditions). GST-mRFP-HA was fused with short peptides related to the KR sequence, and the fusion constructs were analyzed for their ability to localize to the nucleus. As shown in S1 Fig A, GST-mRFP-HA alone was localized in the cytoplasm, whereas the SV40NLS fusion, which served as a positive control, exhibited nuclear localization. Similarly, the KR fusion proteins, KRa and KRb, which were derived from the Xenopus homoeologs of Nemp1, Nemp1a and Nemp1b, exhibited nuclear localization, whereas the KRa(ΔR) fusion did not, indicating that both KRa and KRb sequences function as NLSs (S1 Fig B) and that the first Arg residue of the RKIKXKRAK (X is R or L) motif is required for th [file pone.0127271.s001.tif]

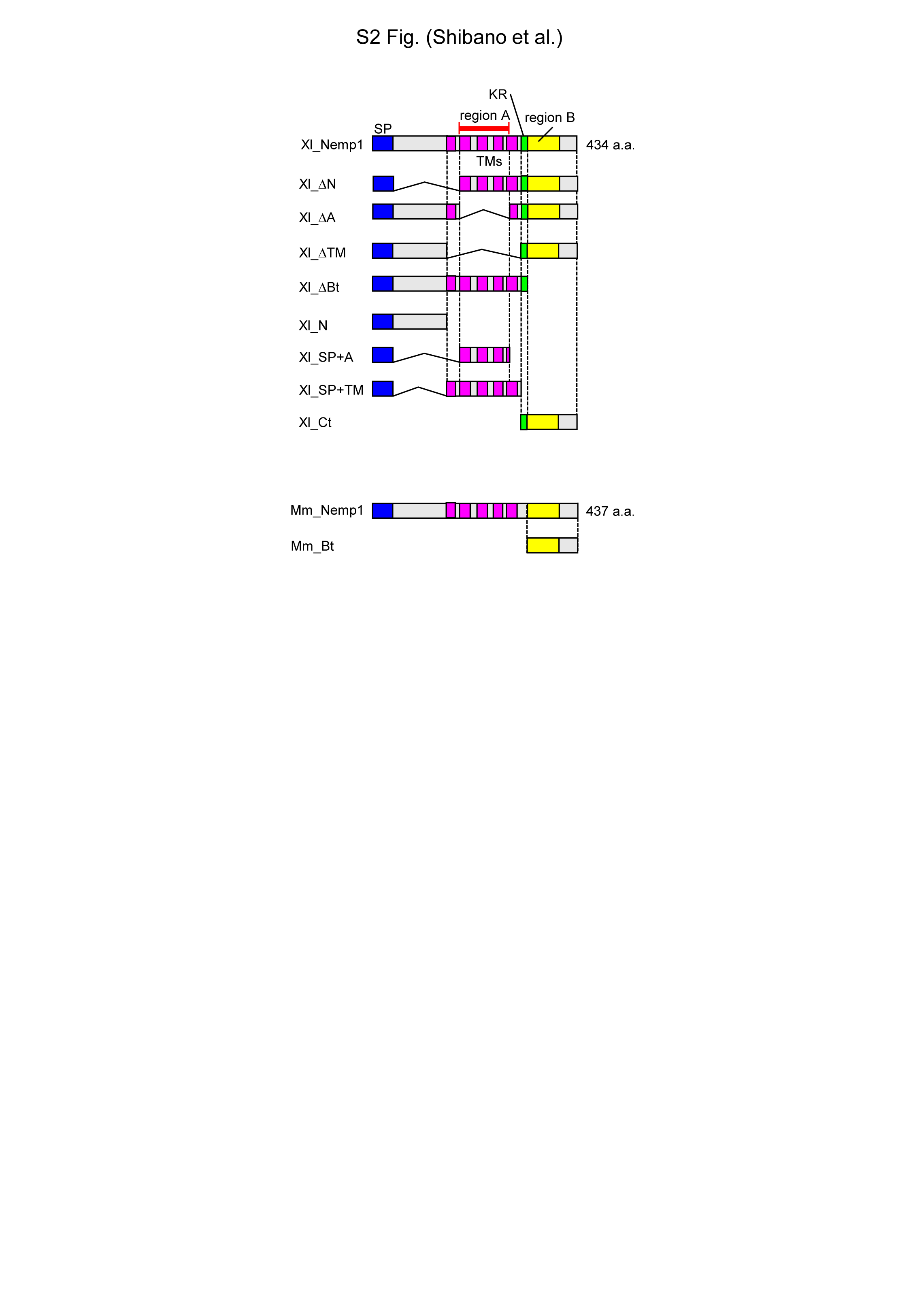

Supplement: S2 Fig — These deletion constructs were used in Fig 2B. Blue, signal peptides (SP); magenta, transmembrane domains (TMs); green, KR sequence; yellow, region B. (TIF) [file pone.0127271.s002.tif]

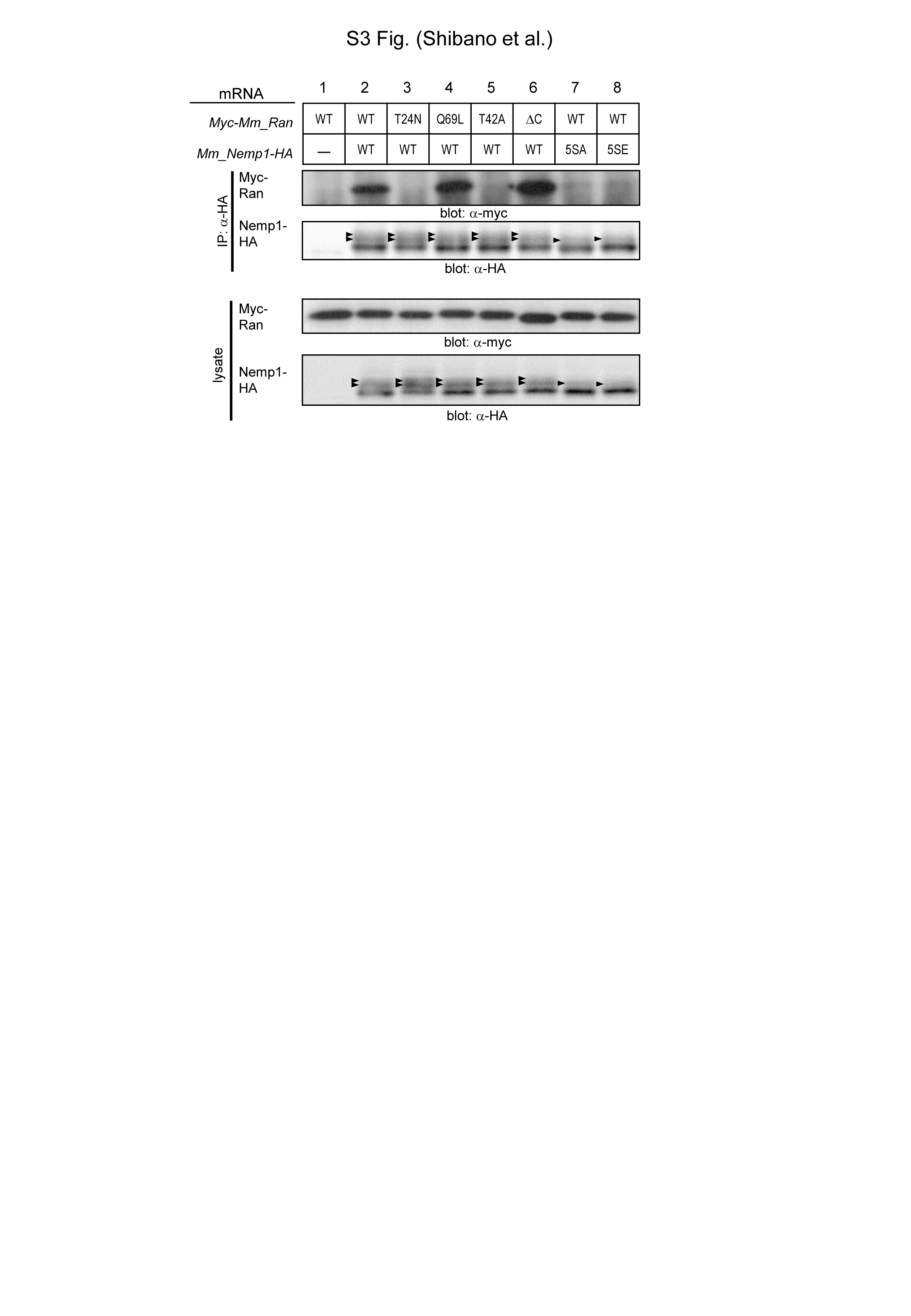

Supplement: S3 Fig — This is the original data for Figs 5A and 6D. mRNA for HA-tagged Mm_Nemp1 or its mutants (5SA, 5SE) was coinjected into Xenopus embryos with mRNA for a Myc-tagged construct of Mm_Ran (WT) or its mutants (T24N, Q69L, T42A, ΔC). Injected embryos were collected at the mid blastula stage (stages 8–8.5) and lysed with lysis buffer B for Co-IP. Black arrowheads, modified forms of Nemp1-HA. Note that WT Nemp1 has two major modified bands (lane 2) and co-expression with Ran(T24N; a GDP form) enhanced these modifications (lane 3). Also note that the upper modified band disappeared in 5SA and 5SE constructs (lanes 7, 8), suggesting that all or some of these five serine residues are involved in modification (phosphorylation) by functioning as either phosphorylation sites or recognition sites or both, and that there are other phosphorylation sites besides there five serine residues. (TIF) [file pone.0127271.s003.tif]

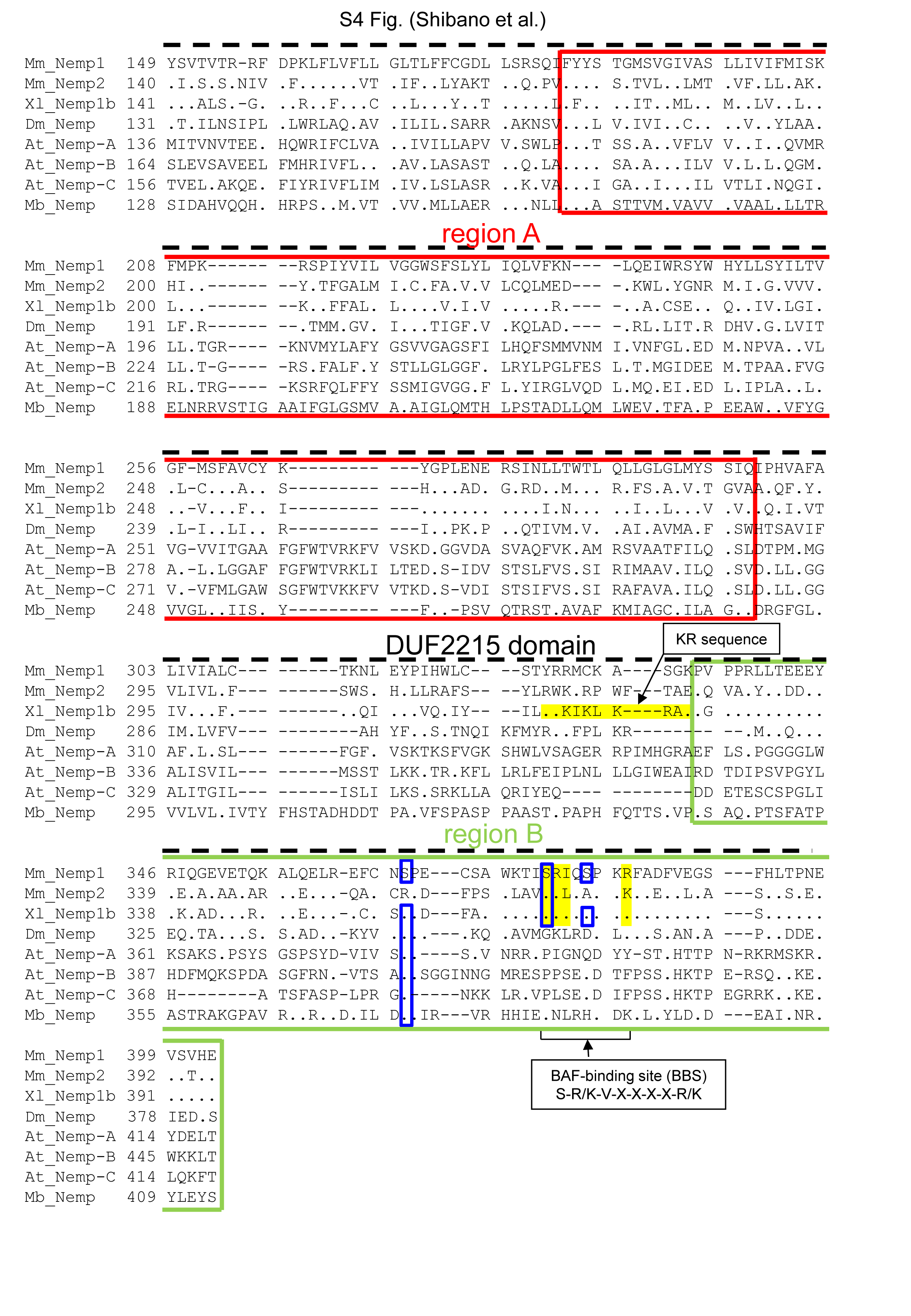

Supplement: S4 Fig — Only region A (red box) and region B (green box) were aligned for Mm_Nemp1, Mm_Nemp2, Xl_Nemp1b, Dm_Nemp, At_Nemp-A, At_Nemp-B, At_Nemp-C, and Mb_Nemp. Dots, identical amino acid residues; hyphens, gaps; dashed line, DUF2215 domain. The KR sequence and BAF binding sites are colored in yellow as indicated. Blue boxes indicates phosphorylation sites in Mm_Nemp1 and the corresponding serine residues in other species. The serine residues corresponding to Ser-366, Ser-376, and Ser380 (but not Ser419, and Ser420) in Mm_Nemp1 are conserved in Xl_Nemp1. BAF binding sites containing Ser380 are conserved in vertebrate Nemp1 and Nemp2, but not in others. At, Arabidopsis thaliana; Dm, Drosophila melanogaster; Mb, Monosiga brevicollis (choanoflagellate); Mm, Mus musculus; Xl, Xenopus laevis. (TIF) [file pone.0127271.s004.tif]

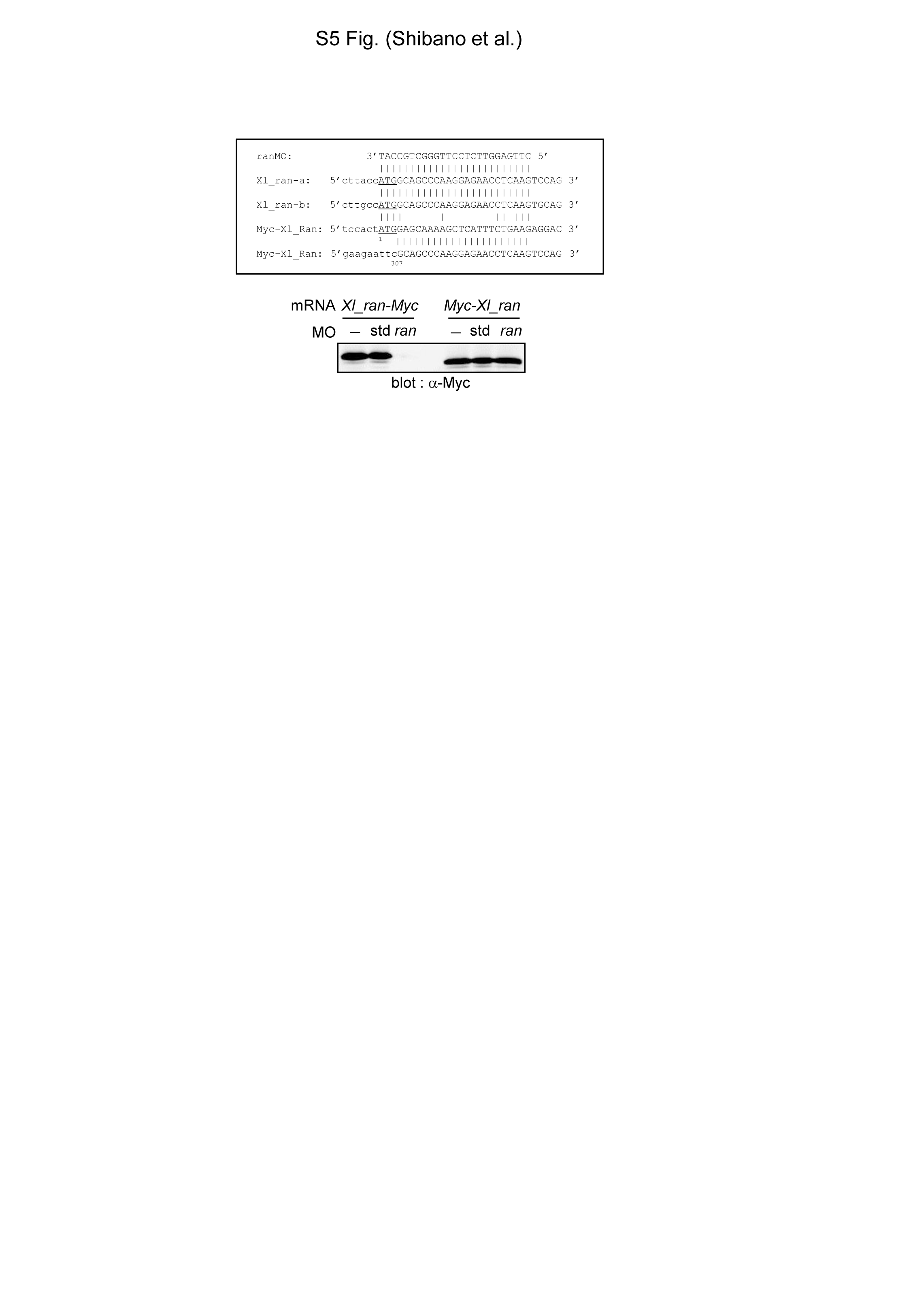

Supplement: S5 Fig — Nucleotide sequences of Xl_ran-a, b mRNAs around the initiation codon (underlined), and ranMO (upper panel). Western blot analysis of Myc-tagged Xl_Ran fusion protein (lower panel). ranMO or stdMO (60 ng) was injected into both blastomeres of two cell stage embryos, and followed by injection with either 200 pg of Xl_Ran-Myc or Myc-Xl_Ran mRNA.-, embryos injected with mRNA alone. (TIF) [file pone.0127271.s005.tif]

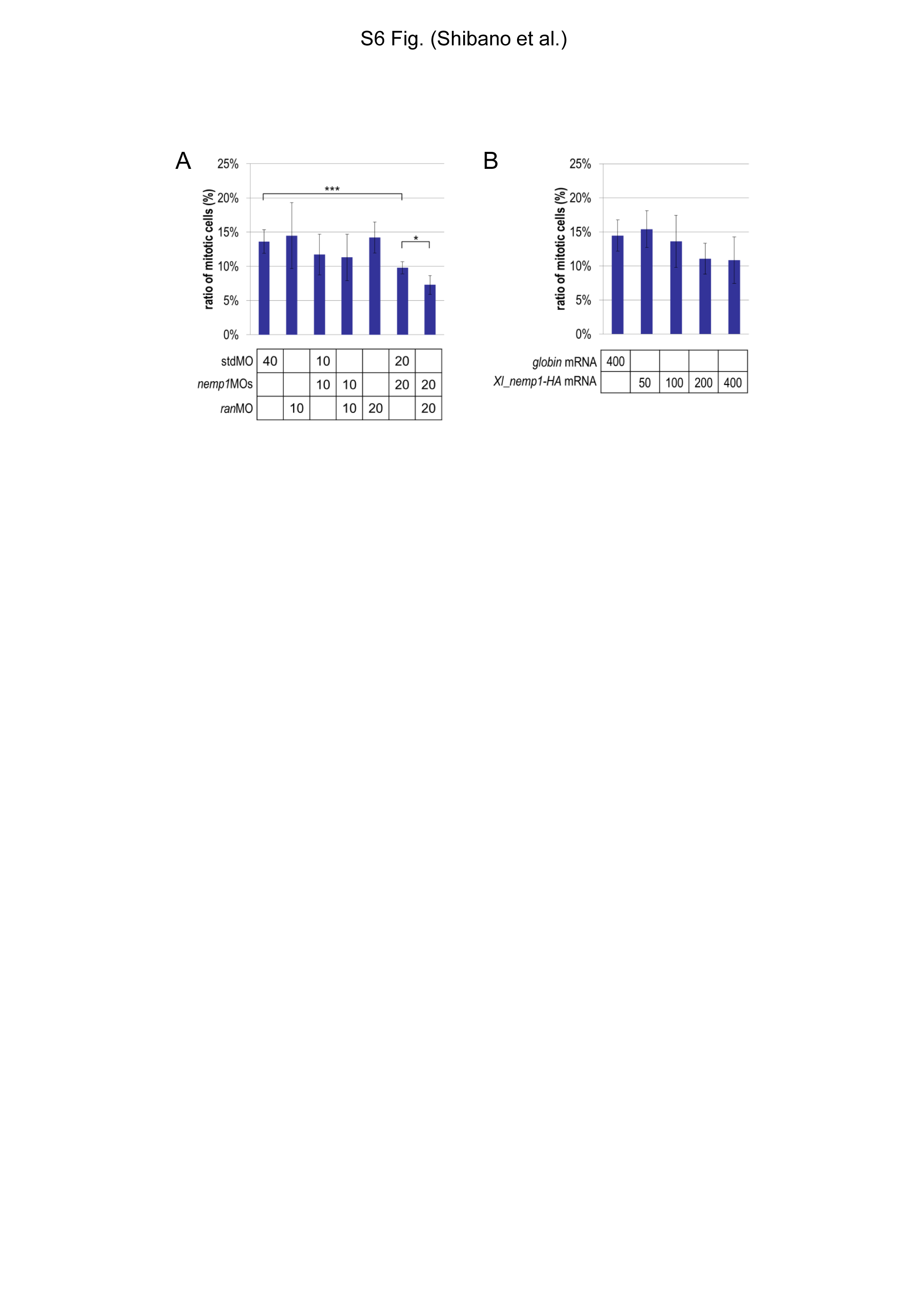

Supplement: S6 Fig — Combinations of injected MOs and mRNAs as well as amounts of MO (ng/embryo) and mRNA (pg/embryo) are as indicated. Experiment conditions are the same as in Fig 8. (A) Reduction of the ratio of mitotic cells by co-knockdown of Nemp1 and Ran. Similar tendencies were obtained from the three experiments and statistically significant differences was observed in one of them. Nuclei stained with DAPI or immunostained for phospho histone H3 were counted in FITC-positive areas. (B) Reduction of the ratio of mitotic cells by overexpression of Nemp1. DAPI-stained nuclei were counted in EGFP-HA positive areas. *, P<0.05; ***, P <0.005; error bars, standard deviation. (TIF) [file pone.0127271.s006.tif]

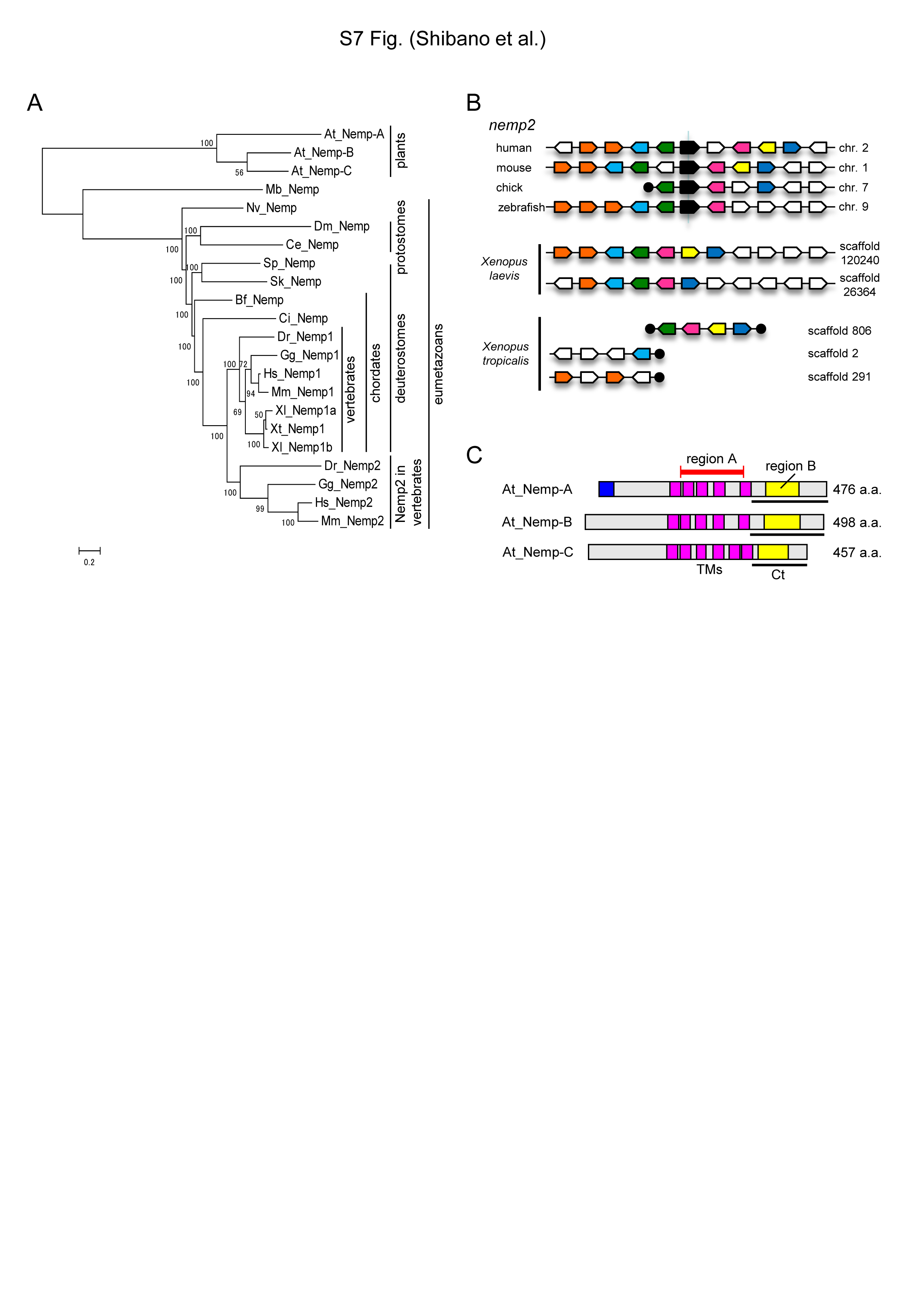

Supplement: S7 Fig — A. Phylogenetic analysis. A phylogenetic tree was constructed by the Maximum Likelihood (ML) method using Treefinder with the protein matrix LG after amino acid sequences of the DUF2215 domain in various organisms were aligned using the ClustalW alignment tool with the Gonnet series protein weight matrix (see S4 Fig) and trimmed using trimAl. Values beside nodes show the number of times that a node was supported in 1000 bootstrap pseudoreplication. Arabidopsis Nemp homologs (At_Nemp-A, B, and C) serve as outgroups. Note that Nemp is evolutionary conserved from metazoans to choanoflagellates to plants, mainly in the terminal part of region A (see S4 Fig). In vertebrates, a Nemp1 homolog, named TMEM194B or Nemp2, is present in the genome databases of zebrafish, chick, mice, and humans. A de novo phylogenetic tree revealed that Nemp1 and Nemp2 form sister groups in vertebrates (not shown), indicating that Nemp2 is the vertebrate paralog of Nemp1. Abbreviations of species and common names are as follows: plant Arabidopsis thaliana (At), Florida lancelet Branchiostoma floridae (Bf), nematode Caenorhabditis elegans (Ce), ascidian Ciona intestinalis (Ci), Drosophila melanogaster (Dm), zebrafish Danio rerio (Dr), chick Gallus gallus (Gg), human Homo sapiens (Hs), choanoflagellate Monosiga brevicollis (Mb), mouse Mus musculus (Mm), sea anemone Nematostella vectensis (Nv), African clawed frog Xenopus laevis (Xl), and western clawed frog Xenopus tropicalis (Xt). Accession numbers of amino acid sequences: Hs_Nemp1, O14524; Mm_Nemp1, Q6ZQE4; Gg_Nemp1, XM_001232566; Xl_Nemp1a, NP_001090391; Xl_Nemp1b, NP_001091224; Xt_Nemp1, NP_001034832; Dr_Nemp1, XP_683418; Hs_Nemp2, A6NFY4; Mm_Nemp2, Q8CB65; Gg_Nemp2, Q5ZJY9; Dr_Nemp2, XP_693037; Bf_Nemp, XP_002585718; Ci_Nemp, AK116477; Sk_Nemp, XP_002741981; Sp_Nemp, XP_001196379; Dm_Nemp, NP_573142; Ce_Nemp, NP_497202; Nv_Nemp, XP_001640959; At_Nemp-A, NM_102639; At_Nemp-B, NM_001037091; At_Nemp-C, NM_114844; Mb_Nemp, XP_001742508. B. Conse [file pone.0127271.s007.tif]

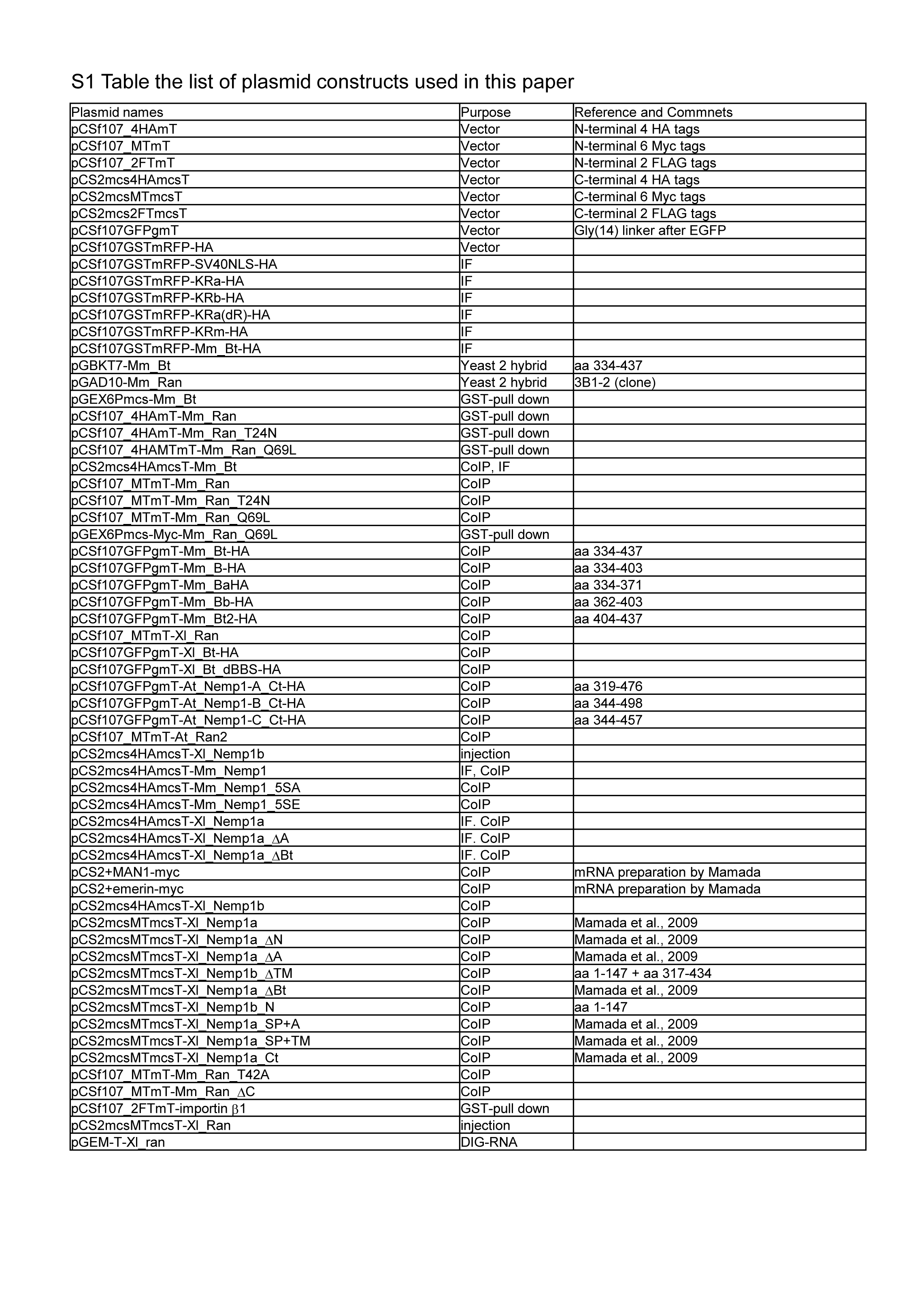

Supplement: S1 Table — (TIF) [file pone.0127271.s008.tif]

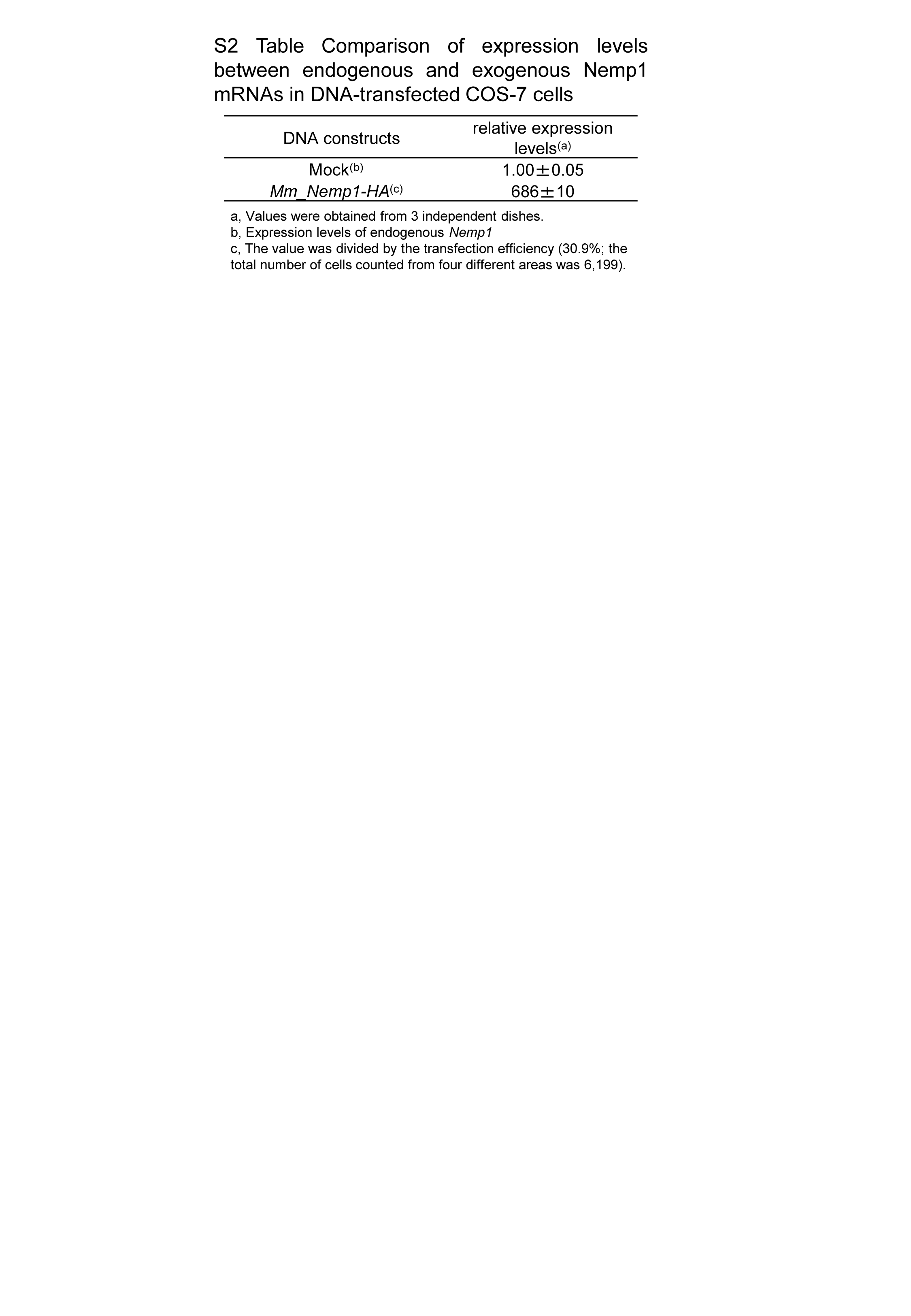

Supplement: S2 Table — (TIF) [file pone.0127271.s009.tif]
